# Femoral Head–Preserving Porous Tantalum Implantation for Osteonecrosis of the Femoral Head: A Systematic Review and Meta-Analysis of Conversion to Total Hip Arthroplasty, Radiographic Progression, and Modes of Failure

**DOI:** 10.3390/medsci14060640

**Published:** 2026-10-05

**Authors:** Tomasz Poboży, Wojciech Konarski

**Affiliations:** 1 Department of Orthopedic Surgery, Medicover Hospital, Aleja Rzeczypospolitej 5, 02-972 Warsaw, Poland; tomasz.pobozy@onet.pl; 2 Medical Rehabilitation Center, Sobieskiego 47D, 05-120 Legionowo, Poland

**Keywords:** osteonecrosis, femoral head, avascular necrosis, tantalum, trabecular metal, joint-preserving surgery, total hip arthroplasty, meta-analysis

## Abstract

**Background:** Porous tantalum implantation is a head-preserving alternative to core decompression in osteonecrosis of the femoral head. Reporting has declined in North America and Europe while continuing elsewhere; outcomes are discordant. Prior syntheses are dated and treated the two endpoints as interchangeable. **Methods:** MEDLINE, Embase, Web of Science, and the Cochrane Library were searched to September 2025. Eligible studies reported head-preserving implantation in ≥5 hips followed ≥12 months, retrieval analyses excepted. The two endpoints were co-primary and pooled separately by random-effects Freeman–Tukey models with Hartung–Knapp adjustment. **Results:** Twenty-two records (946 hips) contributed. Pooled conversion was 20.3% (95% CI 13.5–27.9; I2 = 78.8%; prediction interval 0.3–54.9). Eighteen records (729 hips) reported radiographic progression, pooled at 26.8% (95% CI 16.7–38.2), exceeding conversion within the cohort in twelve of sixteen records with matched denominators (*p* = 0.003). Surgical technique was the only moderator associated with either outcome: rod alone versus an adjunctive procedure gave 27.2% versus 13.9% for conversion (*p* = 0.028) and 37.4% versus 18.4% for progression (*p* = 0.041), surviving adjustment for region but not removal of the one internally controlled cohort or alternative classification of three records. Region, stage, implant generation and follow-up showed no association. It persisted within a single staging system. Retrieval analyses showed minimal ingrowth into necrotic bone. **Conclusions:** Roughly one hip in five proceeds to arthroplasty, and radiographic deterioration is more frequent. Dispersion may relate to whether a substrate for repair was supplied, not to geography or device, but that association is fragile and is offered as a hypothesis.

## 1. Introduction

Osteonecrosis of the femoral head is a disabling condition that characteristically presents in early adulthood; across published surgical cohorts the mean age at treatment lies between 33 and 38 years. It is an important indication for total hip arthroplasty (THA) in this population [1]. Its pathogenesis is multifactorial: corticosteroid exposure, alcohol use, trauma, hemoglobinopathy and idiopathic disease converge on impaired perfusion of the femoral head as a final common pathway. It is the shared endpoint of these processes, producing a segment of necrotic bone in the weight-bearing region and progressive loss of joint congruence [1,2]. The natural history supports early intervention: in a systematic review of sixteen studies comprising 664 asymptomatic hips, 59% progressed to symptoms or collapse, and progression is reported in up to 80% of untreated hips [1,3]. Because that trajectory culminates in arthroplasty at an age associated with a substantial lifetime risk of revision, femoral head-preserving surgery has been pursued for five decades [1,3].

Core decompression remains the reference joint-preserving procedure, with a pooled success rate of approximately 65% across 32 studies, differing significantly by disease stage and by whether the decompression was augmented [4]. It relieves intraosseous pressure and stimulates repair but does not restore mechanical support to the subchondral plate. Porous tantalum implantation was introduced to supply that support: a cylindrical, highly porous construct inserted along the core decompression track into the necrotic segment, combining high volumetric porosity, an elastic modulus approaching that of cancellous bone, and a surface conducive to osseointegration [5,6]. The mechanical support it provided was therefore expected to become biological over time as bone grew into the construct [6].

Clinical experience has not matched this expectation uniformly. The earliest multicenter report described conversion to arthroplasty in 22 of 113 hips [7], and subsequent Western series reported failure rates that led several centers to abandon the procedure [8,9]; the secondary literature describes the device as having been largely phased out of practice in North America and Europe, a shift attributed to suboptimal survivorship and to concern about the consequences of failure [10]. Reporting nevertheless continued, particularly from Asian centers and often with modifications to the original technique, and several later series describe survivorship substantially better than the early Western reports [11,12]. No registry or practice-pattern data quantify that shift, so the inference that use declined rests on the distribution of published reports rather than on direct measurement, and it should be read with that limitation in mind. The literature nevertheless presents a genuine discordance rather than a simple negative result.

Three previous syntheses have addressed the question without resolving it. One favored the rod over bone grafting on functional scores and collapse [13], a second reported conversion to arthroplasty in approximately a quarter of hips but with rates ranging from 6% to 56% across the included studies [14], and a third reported that most hips remained radiographically stable and judged the device a safe alternative [15]. Their conclusions differ in part because they measured different things: conversion to arthroplasty, radiographic stability, or composites of the two. All three are also now dated, the most recent having searched to November 2019 [15], and each preceded a substantial subsequent literature.

That inconsistency points to an unresolved methodological problem. Conversion to arthroplasty and radiographic progression are distinct endpoints occurring at different frequencies within the same cohort, yet previous syntheses have reported one, the other, or a composite, in ways that make their estimates non-comparable; interpretation is further complicated by the coexistence of three staging systems, of which the ARCO classification was itself revised in 2019 [16]. The Ficat, Steinberg, and ARCO systems differ both in the radiographic features that define each grade and in the point at which collapse is recorded, so nominally identical statements about progression are not strictly commensurable between studies. Two bodies of evidence bearing directly on interpretation have also gone unincorporated: implant retrieval analyses, which establish whether the construct osseointegrates within necrotic bone [8,17,18], and studies of conversion arthroplasty after a failed rod, which establish whether the attempt at preservation carries a downstream cost [19,20,21]. Both are small, narrative bodies of evidence that inform interpretation and contribute to no pooled estimate. Accordingly, the primary objective of this systematic review and meta-analysis was to derive contemporary pooled estimates of conversion to THA and of radiographic progression as two separate outcomes that are never combined. The secondary objective was to test pre-specified explanations for between-study heterogeneity, namely surgical technique, disease stage, geographical region, implant generation and duration of follow-up. A third, contextual objective, pursued narratively and without pooling, was to set those estimates against the retrieval and salvage literature, so that the frequency of failure could be interpreted alongside its mechanism and its consequences for the patient.

## 2. Materials and Methods

### 2.1. Protocol and Registration

The review was conducted according to a protocol registered with PROSPERO (CRD420251151228) and is reported in accordance with the PRISMA 2020 statement [22]. The registration covers a program of work examining porous tantalum across orthopedic indications; following completion of the search and screening, the eligible literature was divided by clinical problem into separate reviews sharing one search, one screening process and one extraction dataset, and this division was recorded in an amendment to the registration before any analysis began. The sequence of registration, search, screening, amendment and analysis, with dates, is set out in Supplementary Table S1. The review is accordingly prospectively registered with respect to its search, its eligibility criteria and its outcomes, and retrospectively amended with respect to its scope, since the decision to divide the eligible literature into separate reviews by clinical problem was taken after the search had been run and screening completed. The present review addresses femoral head–preserving implantation in osteonecrosis. Companion reviews addressing fixation in intact host bone, reconstruction of bone loss, and low-volume indications are cited where their scope adjoins the present work.

### 2.2. Eligibility Criteria

Studies were eligible if they reported clinical outcomes in human participants with osteonecrosis of the femoral head of any etiology and at any stage; if the intervention comprised implantation of a porous tantalum device intended to preserve the femoral head, with or without adjunctive procedures; if at least one primary or secondary outcome was reported in extractable form; if at least five hips were included; and if mean follow-up reached at least 12 months or, where only a minimum follow-up was reported, that minimum was at least 12 months. Retrieval and explant analyses were eligible without the follow-up requirement, since the interval to explanation is itself the outcome of interest. Full text in English was required.

Studies were excluded if porous tantalum was used in arthroplasty components rather than as a head-preserving device; if porous tantalum screws were used for femoral neck fracture fixation without an established diagnosis of osteonecrosis; if reconstruction followed oncological resection; if the sole intervention was a porous metal other than tantalum; if the design was preclinical, cadaveric, in vitro or computational without clinical outcomes; if the report was a case report, a series of fewer than five hips, a conference abstract without extractable data, or a narrative review, editorial, letter or commentary; or if no outcome data were extractable. Where cohorts overlapped, the report with the largest sample or the longest follow-up was retained. Overlap was assessed from author lists, institutions, recruitment periods and the described composition of each cohort. Where two reports from the same group could not be shown to be independent but described populations that differed in stage range and in size, both were retained, and a sensitivity analysis removing each, in turn, is reported in Section 3.7.

Pediatric cohorts were eligible but analyzed as a distinct subgroup and excluded in sensitivity analysis. Studies confined to advanced disease were eligible and analyzed as a stage subgroup, since their inclusion was necessary to test whether stage modifies outcome. Studies not meeting the follow-up requirement were excluded from the review in full rather than from individual endpoints, the only exception being retrieval and explant analyses as stated above; one included study met the follow-up requirement with a mean of exactly 12 months but reported neither primary outcome in an extractable form, and therefore contributes functional data only; it is identified as such in tables, and its exclusion from the primary analyses follows from the absence of the outcome data rather than from its follow-up. The registered protocol specified a minimum of ten patients. This review applied a minimum of five hips instead, because hips rather than patients are the unit of analysis throughout this literature and the two thresholds are not interchangeable in a condition that is frequently bilateral; two included reports fall between them. The change is recorded as a deviation in Supplementary Table S1b, and a sensitivity analysis raising the threshold is reported in Section 3.7.

### 2.3. Information Sources and Search

A comprehensive literature search was performed in MEDLINE (PubMed), Embase, Web of Science, and Cochrane Library databases. The search included studies published up to September 2025. No restriction was applied by publication year or study design. The search was supplemented by hand-searching the reference lists of all included articles and of previously published systematic reviews. Full search strategies for each database are provided in Supplementary Table S1a. The strategy registered at inception was executed once for the whole program of work across orthopedic indications, and for the purpose of this analysis, restricted to osteonecrosis of the femoral head. The databases searched and the absence of a publication-year limit differ from the registration, and both differences are recorded in Supplementary Table S1b.

The intervention block was constructed without a core decompression term. Including one improves specificity but excludes the retrieval and salvage literature, which was integral to the review question; specificity was therefore achieved at screening rather than at search.

### 2.4. Study Selection and Data Extraction

Data were extracted into a standardized form covering study identifiers and design, country and number of centers, recruitment period, numbers of patients and hips, demographic characteristics, etiology, classification system and stage distribution, lesion characteristics where reported, implant type and manufacturing method, surgical technique and adjunctive procedures, weight-bearing protocol, follow-up duration and losses, comparator characteristics, and all outcomes. Titles and abstracts were screened by two reviewers working independently; full texts were assessed in the same way, and disagreements were resolved by discussion. No automation tools were used, extracted entries were subsequently recoded into analyzable variables. Data were extracted by one reviewer and checked against the full text by a second.

### 2.5. Outcomes

Co-primary outcomes were conversion to THA, expressed as the proportion of hips converted at longest available follow-up, and radiographic progression, defined as advancement of at least one stage in the classification used by the source study or femoral head collapse as defined by that study. The two were analyzed separately and never pooled together. Because the source studies used three different staging systems, the definition of progression applied in each study is reproduced in Supplementary Table S2, and a sensitivity analysis specified after peer review restricted the pooled estimate to one staging system at a time. Conversion to arthroplasty is the outcome registered at inception as revision of the index implant for any cause, under the name it carries in this indication. Radiographic progression was not among the registered outcomes; it was raised to co-primary status at review level, before any analysis was run, because the two events occur at different frequencies in the same hips and previous syntheses had conflated them. This is a deviation from the registration and is recorded in Supplementary Table S1b. Conversion was taken as reported by each source study. No source study specified a protocol-defined indication for conversion, so in every case the decision reflected some combination of pain, radiographic appearance, patient preference, and surgeon judgment; the consequences for interpretation are considered in Section 4. The three terms used in this literature are distinct and are kept distinct here. Conversion to arthroplasty is an operative event, recorded when the construct was removed and the joint replaced. Radiographic progression is an imaging event, recorded when the hip advanced by at least one grade in the classification used by the source study or when the femoral head collapsed as defined by that study; it does not imply that any operation was performed. Clinical failure is a composite used by some source studies, combining persistent or worsening symptoms with radiographic deterioration, with or without conversion; it was not a primary outcome here and enters the review only in the sensitivity analysis reported in Section 3.7. The two primary outcomes are analyzed separately because they measure different things and occur at different frequencies in the same hips.

Secondary outcomes were the Harris Hip Score (HHS) before operation and at final follow-up; other functional measures; complications; survivorship at defined time points where reported; histological and morphometric retrieval findings; and outcomes of conversion arthroplasty after a failed implant.

### 2.6. Risk of Bias

Randomized trials were assessed with the Cochrane RoB 2 tool [23], non-randomized comparative studies with ROBINS-I [24], and single-arm cohorts and case series with MINORS [25]. ROBINS-I was deliberately restricted to non-randomized studies with a concurrent comparator group, because its domains for confounding and for selection of participants into the study cannot be answered for a single-arm cohort; such cohorts were appraised with MINORS instead, and the two instruments are therefore not applied to the same studies. Retrieval analyses were appraised narratively and excluded from all pooled estimates of incidence, since the specimens constitute a selected sample of failures.

### 2.7. Synthesis

Proportions were pooled with a random-effects model using the Freeman–Tukey double arcsine transformation, with back-transformation at the harmonic mean sample size for presentation. Between-study variance was estimated by restricted maximum likelihood and confidence intervals for pooled estimates were adjusted by the Hartung–Knapp method. Exact binomial confidence intervals were computed for individual studies. Heterogeneity was quantified by τ^2^, I^2^ and Cochran’s Q, and a 95% prediction interval accompanies each pooled estimate.

Pre-specified subgroup analyses examined surgical technique, disease stage, geographical region, implant generation, study design, and risk of bias, tested by Q between groups. Surgical technique was classified from the procedural description of each source study as rod alone, meaning that no material was placed within the femoral head; as rod with removal of necrotic bone and impaction of graft or bone substitute into the resulting cavity; or as rod with biological augmentation, meaning the addition of marrow aspirate, cultured cells or a vascularized graft. Curettage of the reamed tract without filling of the cavity was classified as rod alone, since the distinction of interest is whether a substrate for repair was supplied rather than whether necrotic bone was disturbed. One study reported outcomes separately by technique; in the technique analysis its two arms were entered in place of the pooled record, so that no cohort contributed twice. A cohort was classified as advanced stage when more than half of its hips were at stage III or higher at implantation in the classification used by the source study, as early stage when more than half were at stage I or II, and as mixed otherwise.

Meta-regression examined mean follow-up duration, advanced stage and geographical region as covariates, with Knapp–Hartung standard errors. A bivariable model examined technique with region held constant. The proportion of between-study variance explained was computed as the relative reduction in τ^2^. Because mean follow-up varied more than fivefold between records, conversion was additionally analyzed as an incidence rate. Hip-years of observation were computed as the number of hips multiplied by mean follow-up, and log rates were pooled by the same random-effects model, with a continuity correction of half an event applied to the single record reporting no conversions.

Sensitivity analyses comprised leave-one-out estimation; exclusion of studies at high risk of bias; exclusion of the pediatric cohort; restriction to studies with mean follow-up of at least 24 months; and re-analysis under the broader clinical-failure definition used by some source studies. Further analyses, specified after peer review and therefore post hoc, restricted the pooled estimate of radiographic progression to one staging system at a time and repeated the surgical-technique comparison within the largest single-system stratum; raised the minimum cohort size progressively from five to twenty hips; removed, in turn, each of the two reports whose cohorts might overlap; and restricted the paired comparison of the two endpoints to records in which the same hips contributed to both. Small-study effects were examined by funnel plot and, where at least ten records contributed to a pooled estimate, by Egger’s regression test; in accordance with Cochrane guidance the test was not applied to smaller sets, which excludes every subgroup analysis reported here. Even where the test was applied, the number of records is close to that threshold, so a negative result carries little evidential weight and is not treated as evidence that small-study effects are absent.

The HHS was synthesized descriptively rather than pooled as a summary of functional benefit. Change from baseline is reported study by study together with the range of study-level effects and the between-study dispersion; a standardized mean change and a raw mean change were computed and are quoted only to document that dispersion. The pre–post correlation required for the standardized mean change was imputed at 0.5 and varied between 0.3 and 0.7 in sensitivity analysis. Studies reporting medians and interquartile ranges were not converted to means and standard deviations, because the conversion assumes an approximately symmetrical distribution that could not be verified in these reports.

Retrieval and salvage evidence was synthesized narratively. Certainty of evidence was rated with GRADE, with a starting level of low for a body of evidence dominated by single-arm observational studies.

Analyses were performed in Python 3 using purpose-written implementations of the Freeman–Tukey pooling, REML variance estimation, and meta-regression procedures.

## 3. Results

### 3.1. Study Selection and Characteristics

Twenty-six reports met the eligibility criteria (Figure 1). Two of these randomized or allocated participants to two distinct interventions and reported each separately; they are entered as two records apiece: one compared two tantalum devices head to head [26], and one compared implantation of the rod alone against implantation combined with impaction grafting [12]. Twenty-two records from twenty publications, comprising 946 hips, therefore contributed to the primary endpoint of conversion to arthroplasty. One further study with 12 months of follow-up and no reported conversions [27] was excluded from that endpoint by protocol and contributed functional data only. Three retrieval analyses [8,17,18] and three studies of conversion arthroplasty after a failed implant [19,20,21] were synthesized narratively.

Study characteristics are summarized in Table 1. Fourteen records derived from Asian centers and eight from North America, Europe or Australia. Three were randomized, five were non-randomized comparative studies, and fourteen were single-arm prospective or retrospective series. Mean follow-up ranged from 18.4 to 100.1 months. Seventeen records reported the number of patients as well as the number of hips; in these, the ratio of hips to patients ranged from 1.00 to 1.37, with a median of 1.16, and bilateral disease was reported in proportions ranging from none of the patients in one pediatric cohort to all of them in one early series. Hips rather than patients are the unit of analysis throughout, as in every source study. Cohorts were predominantly early-stage in twelve records, predominantly advanced in two, and mixed in eight. A first-generation wrought device was used in sixteen records and a later-generation or additively manufactured device in four; the device was not specified in two.

**Table 1:** Characteristics and risk of bias of all 26 included publications, grouped by analytical domain.

Record	Region	Design	Hips	FU (mo)	Stage	Technique	Device	THA n/N	Progression n/N	RoB
Tsao 2005 [7]	West	Multicenter	113	48.0	Steinberg I–II	Rod alone	Zimmer	22/113	NR	Moderate
Veillette 2006 [28]	West	Retrospective	58	24.0	Steinberg I–III	Rod alone	Zimmer TM	9/58	16/58	Moderate
Nadeau 2007 [29]	West	Prospective	18	23.0	Steinberg III–IV	Rod alone	Zimmer	9/18	NR	High
Shuler 2007 [9]	West	Prospective	22	39.0	UPenn I–II	Rod alone	Zimmer TM	3/22	3/22	High
Floerkemeier 2011 [30]	West	Retrospective	23	18.7	ARCO I–II	Rod alone	Zimmer OII	13/23	1/10	Moderate
Liu ZH 2014 [31]	Asia	Retrospective	138	38.5	ARCO II–III	Rod + graft	Zimmer	43/138	47/138	Moderate
Liu Y 2015 [32]	Asia	Prospective	57	44.8	Steinberg I–IVA	Rod + graft	Zimmer TM	11/57	17/57	Moderate
Miao 2015 [33]	Asia	Randomized	36	25.6	Steinberg I–II	Rod alone	NR	8/36	8/36	Moderate
Osman 2015 [34]	West	Prospective	20	24.0	Steinberg II–III	Rod alone	NR	5/20	NR	Mod–High
Pakos 2015 [11]	West	Retrospective	58	60.0	Steinberg II–IIIa	Rod + biol.	Zimmer TM	4/58	7/58	Moderate
Zhao D 2015 [35]	Asia	RCT	31	64.4	ARCO IIIc–IV	Rod + biol.	Zimmer TM	5/31	8/31	High
Liu Y 2016 [36]	Asia	Prospective	52	48.2	Steinberg I–II	Rod + graft	Zimmer TM	8/52	NR	Moderate
Zhang X 2016 [18]	Asia	Prospective	14	19.0	ARCO II–III	Rod alone	TaBw01	5/14	6/13	High
Hu 2018 [37]	Asia	Case series	29	64.8	ARCO I–II	Rod + graft	Zimmer TM	2/29	4/29	Moderate
Huang W 2019 (Runze) [26]	Asia	RCT	21	38.2	ARCO I–IIB	Rod + graft	Runze	1/21	0/21	Moderate
Huang W 2019 (Zimmer) [26]	Asia	RCT	20	39.5	ARCO I–IIB	Rod + graft	Zimmer	0/20	0/20	Moderate
Fang 2020 [38]	Asia	Comparative	41	33.5	Ficat I–II	Rod alone	Zimmer TM	5/41	26/41	High
He 2020 [39]	Asia	Retrospective	40	100	ARCO II–III	Rod + graft	Zimmer TM	12/40	22/40	Moderate
Zhang Y 2021 [40]	Asia	Multicenter	52	85.7	ARCO I–II	Rod alone	Zimmer	24/52	28/52	Moderate
Hussaini 2021 [41]	West	Retrospective	9	18.4	Ficat I–III	Rod alone	Zimmer	0/9	4/9	High
Huang J 2024 (rod) [12]	Asia	Comparative	44	32.7	ARCO IIa–IIc	Rod alone	TaBw01	21/44	24/44	Moderate
Huang J 2024 (graft) [12]	Asia	Comparative	50	32.7	ARCO IIa–IIc	Rod + graft	TaBw01	7/50	9/50	Moderate
Included; contributing functional outcome data only										
Peng 2020 [27]	Asia	RCT	38	12.0	ARCO I–II	Rod + graft	Zimmer TM	NR	NR	Moderate
Retrieval analyses										
Tanzer 2008 [8]	West	Retrieval	15	13.4	Steinberg II	Rod alone	Zimmer OII	—	14/15	High
Zhang X 2016 [18]	Asia	Retrieval	4	10.2	ARCO II–III	Rod alone	TaBw01	—	—	High
Fernandez-Fairen 2012 [17]	West	Retrieval	5	24.0	Steinberg II	Rod alone	NR	—	NR	High
Conversion arthroplasty after failure										
Cheng 2018 [19]	Asia	Comparative	39	88.8	NR	—	—	—	—	Moderate
Ma 2020 [20]	Asia	Matched cohort	34	64.1	ARCO III–IV	—	—	—	—	Moderate
Zhao F 2020 [21]	Asia	Comparative	65	76.8	ARCO III–IV	—	—	—	—	Moderate

Two publications reporting distinct interventions separately are entered as two records apiece, giving 22 records that contribute to the primary endpoint. NR, not reported; em dash, not applicable to that domain. Risk of bias was assessed with RoB 2 for randomized cohorts, ROBINS-I for non-randomized comparative studies and MINORS for single-arm series; only the overall judgement is shown. Technique: rod alone; rod with removal of necrotic bone and impaction grafting (rod + graft); rod with biological augmentation (rod + biol.). The first block comprises the 22 records contributing to the primary endpoint. Peng 2020 [27] met the eligibility criteria but had 12 months of follow-up and reported no conversions; it was excluded from the primary endpoint by protocol and contributed functional data only. Retrieval and salvage studies are listed separately because they inform interpretation rather than support estimation and are excluded from all pooled estimates; sample size denotes explanted specimens for retrieval analyses and hips undergoing conversion arthroplasty for the salvage domain. Zhang X 2016 [18] reports a prospective cohort together with a retrieval analysis of the explanted rods and therefore appears in both the efficacy and the retrieval block; it is a single publication, and the two rows describe the same patients. Its hips are counted once in the pooled estimates and its explants once in the retrieval synthesis.

### 3.2. Risk of Bias

Fifteen records were judged at moderate risk of bias, one at moderate to high, and six at high (Table 1). The dominant concerns in the single-arm literature were incomplete reporting of consecutive recruitment, absence of a prospectively defined endpoint, and losses to follow-up not accounted for. The randomized cohorts raised concerns regarding allocation concealment and the absence of blinded outcome assessment, the latter material given that radiographic progression is an assessor-determined endpoint.

### 3.3. Conversion to Total Hip Arthroplasty

Overall, 217 of 946 hips were converted to arthroplasty, a crude proportion of 22.9%. The pooled proportion was 20.3% (95% CI 13.5–27.9), with substantial heterogeneity (tau-squared = 0.027, I-squared = 78.8%, Q = 99.1, df = 21, *p* < 0.001) and a 95% prediction interval of 0.3–54.9% (Figure 2). Individual estimates ranged from 0% [26,41] to 56.5% [30].

The prediction interval indicates that a future series of this procedure, performed under conditions comparable to those already reported, could plausibly yield a conversion rate anywhere from negligible to more than half. The analyses that follow were directed at the sources of this dispersion.

Because mean follow-up ranged from 18.4 to 100.1 months, conversion was also analyzed as an incidence rate. The 22 records contributed 3548 hip-years of observation, a crude rate of 6.12 conversions per 100 hip-years and a pooled random-effects rate of 6.48 per 100 hip-years (95% CI 4.29–9.81; I-squared 82.3%; 95% prediction interval 1.17–35.81). Heterogeneity was of the same order as in the analysis of proportions, and the ordering of records by rate corresponded closely to their ordering by proportion (Spearman rho = 0.84, *p* < 0.001), so the two analyses identify the same records as extreme. The records with the shortest follow-up and the highest event fractions moved to the top of the rate ordering, at 36.3 and 26.1 conversions per 100 hip-years [29,30], and those with the longest follow-up and fewest events to the bottom, at 1.3 and 1.4 [11,37]. The incidence analysis therefore does not overturn the analysis of proportions, but it makes explicit that the pooled proportion is an average taken over unequal observation periods and is not a survivorship estimate at any particular time point. No group of records reported survivorship at time points common enough for a pooled time-specific estimate to be constructed, which is why none is given.

### 3.4. Radiographic Progression

Eighteen records comprising 729 hips reported radiographic progression or collapse, pooled at 26.8% (95% CI 16.7–38.2; tau-squared = 0.046, I-squared = 85.6%; 95% prediction interval 0.1–73.9) (Figure 3).

Eighteen records reported both endpoints, but in two of them, radiographic follow-up was incomplete, so that the denominators for the two endpoints differ and a paired comparison is not admissible [18,30]. Among the sixteen records in which the same hips contributed to both endpoints, radiographic progression exceeded conversion to arthroplasty within the cohort in twelve, the two were equal in three, and conversion exceeded progression in one (median 26.7% versus 14.8%; Wilcoxon signed-rank *p* = 0.003). The gap was wide in several series: 63.4% against 12.2% [38], 55.0% against 30.0% [39], and 53.8% against 46.2% [40].

Radiographic progression was defined against three different staging systems, and the definition applied by each source study is reproduced in Supplementary Table S2. In a post hoc analysis restricted to one system at a time, the pooled proportion was 25.4% (95% CI 10.8–43.1; k = 11, 149 of 448 hips; I-squared 88.0%) among studies using the ARCO classification, 22.5% (95% CI 10.5–37.3; k = 4, 48 of 209 hips; I-squared 54.5%) among those using the Steinberg classification and 60.2% (95% CI 45.1–74.5; k = 2, 30 of 50 hips; I-squared 3.3%) among the two using the Ficat classification; the single record using the University of Pennsylvania system (3 of 22 hips) was not pooled. The difference between systems was statistically significant (Q between groups = 19.58, df = 2, *p* < 0.001), but it is generated entirely by the two Ficat records, which are the two smallest in the dataset (41 and 9 hips) and both of which implanted the rod alone, so that staging system and surgical technique are completely confounded in that stratum. Within the ARCO stratum, which is the largest and contains both techniques, the technique contrast persisted: 44.1% (95% CI 14.8–75.7; k = 4) after the rod alone against 17.7% (95% CI 2.8–40.2; k = 7) with an adjunctive procedure (Q between groups = 4.13, df = 1, *p* = 0.042). The surgical-technique association is therefore not an artifact of differing definitions of progression, although the between-system comparison itself should not be read as a property of the classifications.

The endpoint itself is composite. Progression was recorded when a hip advanced by at least one grade in the classification used by the source study or when the femoral head collapsed as defined by that study, and no source report gave the two components separately in a form that allowed them to be disaggregated. Stage advancement without collapse and collapse itself are not equivalent clinical events, and the pooled value of 26.8% combines them in proportions that differ between records and cannot be recovered. That, together with the three staging systems and the absence of blinded assessment in all but a few reports, is the reason the certainty rating for this outcome is downgraded for indirectness, and the reason the pooled value should be read as a summary of a heterogeneous endpoint rather than as an incidence of femoral head collapse.

### 3.5. Surgical Technique

Surgical technique was the only pre-specified moderator associated with either primary outcome (Figure 4). For conversion to arthroplasty, implantation of the rod alone was associated with a pooled proportion of 27.2% (95% CI 16.3–39.5; k = 12) against 14.7% (95% CI 6.1–25.8; k = 8) where necrotic bone was curetted and the defect grafted, and 10.2% (95% CI 2.8–20.9; k = 2) where biological augmentation was added (Q between groups = 6.44, df = 2, *p* = 0.040). Collapsing the two adjunctive categories gave 27.2% against 13.9% (95% CI 7.1–22.4; Q between groups = 4.86, df = 1, *p* = 0.028).

The same contrast held for radiographic progression: 37.4% (95% CI 22.6–53.3; k = 9) after the rod alone against 18.4% (95% CI 6.3–34.6; k = 9) with an adjunctive procedure (Q between groups = 4.19, *p* = 0.041).

In a bivariable meta-regression with geographical region held constant, adjunctive technique remained associated with lower conversion (beta = −0.209 on the transformed scale, SE 0.085, *p* = 0.023), and the model accounted for approximately 23% of between-study variance, with residual tau-squared falling from 0.027 to 0.021. Region contributed nothing in the same model (*p* = 0.296).

The most direct evidence comes from the study that compared techniques within a single cohort [12]. Conversion occurred in 21 of 44 hips treated by rod alone and 7 of 50 treated by rod with impaction grafting (47.7% versus 14.0%; risk ratio 3.41, 95% CI 1.60–7.24; *p* < 0.001; absolute risk difference 33.7 percentage points). Radiographic progression in the same cohort occurred in 24 of 44 against 9 of 50 (54.5% versus 18.0%; risk ratio 3.03, 95% CI 1.58–5.81; *p* < 0.001; absolute risk difference 36.5 percentage points).

Three qualifications must be stated, and the first is decisive for how strongly this finding should be read. When that internally controlled cohort is removed altogether, the between-group difference is no longer conventionally significant for either endpoint (conversion *p* = 0.069; radiographic progression *p* = 0.103), and if both split publications are instead re-pooled as single records, the difference attenuates further (*p* = 0.211). The aggregate signal therefore depends materially on one study. Second, technique is unevenly distributed across regions: seven of eight Western records used the rod alone against five of fourteen Asian records (Fisher exact *p* = 0.031), so technique and region are confounded, although region alone shows no association and does not survive adjustment. Third, all comparisons are between study arms rather than randomized contrasts, with the single exception noted above. Fourth, the choice of technique is not random. It is likely to be influenced by disease stage, by lesion size and location, by symptom severity, and by surgeon preference, none of which could be adjusted for at the record level, so confounding by indication cannot be excluded and may act in either direction. The technique effect is consistent in direction across four analyses and two endpoints, but is not established by any of them individually, and is best regarded as a hypothesis for prospective testing.

### 3.6. Moderators Showing No Association

Geographical region was not associated with conversion (Asia 20.1%, 95% CI 12.0–29.5; West 20.7%, 95% CI 7.0–38.6; Q between groups = 0.02, *p* = 0.898).

No association was found for disease stage (early 19.4%, advanced 31.0%, mixed 19.4%; *p* = 0.780), implant generation (first generation 19.1%, later generation 23.4%; *p* = 0.813), or risk of bias (*p* = 0.874). Meta-regression showed no relationship between conversion and mean follow-up (beta = −0.00015, SE 0.00198, *p* = 0.939; Figure 5), advanced stage (*p* = 0.410), Western origin (*p* = 0.892), or use of a later-generation device (*p* = 0.676).

The null result for implant generation is reinforced by the only head-to-head comparison in the dataset. In a randomized trial of two tantalum devices implanted by the same technique at the same center [26], conversion occurred in 1 of 21 hips with one device and 0 of 20 with the other (Fisher exact *p* = 1.00).

Conversion is a cumulative endpoint, so an association with follow-up duration would be expected on ordinary grounds; none were detected across a range of mean follow-up from 18.4 to 100.1 months.

Because the absence of an association in meta-regression does not establish that follow-up duration is without influence, conversion was also estimated within bands of comparable follow-up. Records with mean follow-up below 30 months gave a pooled proportion of 27.4% (95% CI 10.4–48.2; k = 7), those between 30 and 48 months 16.5% (95% CI 5.8–30.9; k = 8), and those of 48 months or more 19.0% (95% CI 8.3–32.6; k = 7), with no difference between bands (Q between groups = 1.58, df = 2, *p* = 0.455). Restricting to the twelve records with mean follow-up between 24 and 48 months, the narrowest band containing a usable number of records, gave 18.0% (95% CI 10.7–26.6) with I-squared of 74%. The corresponding bands for radiographic progression were 26.7%, 23.1%, and 30.8% (*p* = 0.848). Two observations follow. Heterogeneity remains substantial within every band, so unequal observation time is not the source of the dispersion; and the ordering of the bands is not monotonic, the shortest-follow-up band giving the highest conversion proportion, which is the opposite of what a purely cumulative process would produce and points instead to differences in case mix and technique between the records that report short and long follow-up. Neither analysis shows that duration of follow-up is irrelevant, and the pooled proportions remain averages over unequal observation periods.

These are null results in analyses of limited power rather than demonstrations of equivalence. With 22 records, unequal subgroup sizes and residual between-study variance of this magnitude, a moderate association would not reliably have been detected, and none of these findings should be read as evidence that the moderator concerned is unimportant.

The null result for disease stage is limited by the coding available. Only three studies reported conversions broken down by stage, so stage could be modelled only at the level of the predominant stage of each cohort. The influence of stage therefore could not be adequately assessed, and the null result reflects insufficient power rather than evidence of no effect: twelve records were classified as predominantly early stage, eight as mixed and only two as predominantly advanced, so the advanced-stage estimate rests on two cohorts. Differences in the stage distribution between records are themselves a likely contributor to the observed heterogeneity rather than a factor that can be excluded as irrelevant.

One moderator not pre-specified deserves mention because it is unlikely to be causal. Randomized cohorts reported a lower pooled conversion proportion than non-randomized ones (5.7%, 95% CI 0.1–38.4, k = 3, against 22.9%, 95% CI 15.7–30.8, k = 19; Q between groups = 5.35, *p* = 0.021). The randomized subgroup comprises three small cohorts with six events in total, two of which are the arms of a single trial [26]; when those arms are re-pooled the difference is no longer significant (*p* = 0.110). The finding most plausibly reflects short follow-up and small size among the randomized cohorts rather than any property of randomization and is reported for completeness.

### 3.7. Sensitivity Analyses

The pooled estimate was stable across all pre-specified sensitivity analyses (Table 2): 20.8% when the two split publications were re-pooled as single records, 20.5% restricting to records at moderate risk of bias, 21.2% excluding the pediatric cohort, 18.3% restricting to records with mean follow-up of at least 24 months, and 21.4% under the broader clinical-failure definition. Leave-one-out estimates ranged from 19.0% to 21.6%, with no single record altering the pooled value by more than 1.3 percentage points. Heterogeneity remained substantial throughout (I-squared 76–83%). Three further analyses were added in response to peer review. Raising the minimum cohort size from 5 hips to 10, 15, and 20 gave pooled proportions of 21.2%, 20.7%, and 19.7%, and the surgical-technique contrast remained significant at the highest threshold (27.4% against 13.9%; *p* = 0.032); restricting radiographic progression to records of at least 20 hips gave 26.1% against 26.8% in the base case. Removing Liu Y 2016 [36], whose cohort might overlap with that of Liu Y 2015 [32], gave 20.5%, and removing Liu Y 2015 instead gave 20.3%, with the technique contrast unaffected in both (*p* = 0.032). No record reported the recruitment period, so independence of those two cohorts could not be established directly; they differ in stage range, in size and in the presence of a comparator arm, and the pooled estimate does not depend on the decision either way.

The technique classification is the single variable on which the principal subgroup finding rests, and three records sit close to its boundary; what each source study reported about the handling of the necrotic segment is set out in Supplementary Table S3. Two records classified as rod alone describe curettage or scraping of the reamed tract with nothing placed in the resulting cavity [38,40], and one classified as adjunctive describes clearance of the lesion without stating that any material was placed [37]. Reclassifying the two curettage records as adjunctive gave 27.0% against 15.9% for conversion (*p* = 0.090) and 30.3% against 24.9% for radiographic progression (*p* = 0.54). Reclassifying the third record as rod alone gave 25.2% against 14.7% (*p* = 0.082) and 34.5% against 18.9% (*p* = 0.109). Applying all three changes together gave 24.7% against 16.7% (*p* = 0.218) and 27.7% against 26.0% (*p* = 0.818). The direction of the association is preserved for conversion under every coding examined and for radiographic progression under all but the most adverse, but its statistical significance is not preserved under any of them. The pre-specified classification was applied before this question was raised and is retained as the primary analysis, but the finding must be read as dependent on how a small number of procedural descriptions are classified, and this is reflected in the certainty rating.

**Table 2:** Sensitivity analyses for conversion to total hip arthroplasty.

Analysis	k	Pooled % (95% CI)	I^2^
Base case	22	20.3 (13.5–27.9)	79%
Split publications re-pooled	20	20.8 (14.2–28.3)	78%
Moderate risk of bias only	15	20.5 (12.0–30.3)	83%
Excluding the paediatric cohort	21	21.2 (14.4–28.8)	79%
Mean follow-up ≥24 months	18	18.3 (12.3–25.1)	77%
Broader clinical-failure definition	22	21.4 (14.0–29.9)	81%
Asian records only	14	20.1 (12.0–29.5)	79%
Western records only	8	20.7 (7.0–38.6)	80%

Egger’s regression gave no evidence of small-study effects (intercept -0.94, SE 1.52, t = −0.62, df = 20, *p* = 0.540; Figure 6). The test was applied only to the two primary pooled estimates, each of which drew on more than 10 records, and not to any subgroup, where fewer than 10 records were available.

**Figure 6:**
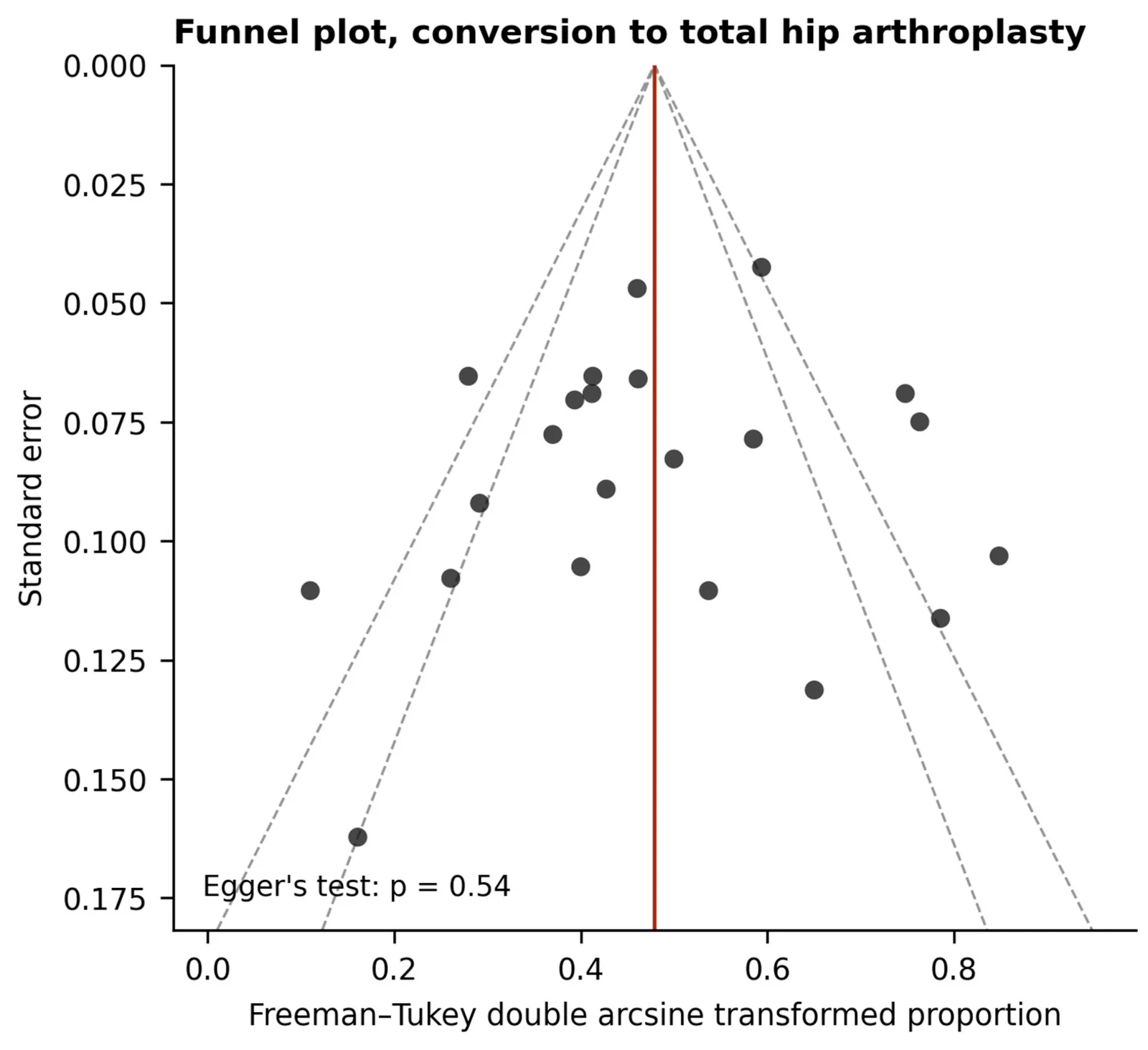
Funnel plot of the Freeman–Tukey transformed proportion against standard error, with pseudo 95% and 99% confidence limits.

### 3.8. Complications

Complications were reported narratively by most records and in a standardized classification by none, so no pooled estimate is offered. Deep infection requiring removal of the construct, staged conversion or both was reported in four hips in four series [18,28,32,40], and superficial wound infection in two [11,28]. Fracture occurred in two hips: a periprosthetic fracture four months after implantation, following a fall from standing height [29], and a subtrochanteric fracture after trauma [30], both treated by fixation. Trochanteric bursitis was reported in four hips [28,32]. Two problems of device handling were described: damage to the driver end of the implant during seating in four hips of the earliest multicenter series, corrected by a change in technique [7], and one perforation of the articular surface with a mis-sized rod, converted at eight months [26]. Two hips in one cohort required reoperation for nonunion of the greater trochanteric osteotomy used to gain access for impaction grafting, which is a complication of the adjunctive procedure rather than of the implant [12]. Six records stated explicitly that no complication occurred [9,34,35,37,38,39]. No record reported fracture, loosening or migration of an implanted construct, and the largest retrieval series recorded no gross migration in any of fifteen specimens despite minimal ingrowth [8].

### 3.9. Functional Outcome

Nine cohorts reported the HHS with dispersion measures at both time points. Because heterogeneity was extreme, these data are reported descriptively, and no pooled value is offered as a summary of functional benefit. Study-level standardized effects ranged from −0.35 to +8.32. For completeness, a random-effects synthesis gives a raw change of 19.4 points (95% CI 8.7–30.1) and a standardized mean change of 2.64 (95% CI 0.42–4.85), with I-squared of 97–99% and a 95% prediction interval for the raw change spanning from −14.6 to 53.4 points; these figures are quoted to document the dispersion around them and for no other purpose. Varying the imputed pre–post correlation between 0.3 and 0.7 left both values unchanged to two decimal places, because the weights are governed by the between-study rather than the within-study variance. Mean scores at final follow-up ranged from 59.9 to 95.8 with a median of 71.1, and only one of the nine cohorts exceeded 90, so a ceiling effect is unlikely to account for the dispersion. One cohort reported deterioration in mean score [40], and the largest effects came from cohorts with the lowest baseline scores [26,35], a pattern consistent with regression to the mean in the absence of control arms. Two further studies reported the score without dispersion measures [7,34], one reported medians with interquartile ranges [39], and one used the Merle d’Aubigné-Postel score [11]; all four were excluded from these estimates by protocol. Converting the reported medians by the method of Wan and colleagues and adding that study raised the standardized mean change from 2.64 to 4.21 (95% CI −0.10 to 8.51) and the raw change from 19.4 to 21.1 points, which is a further reason for treating this synthesis as descriptive.

Two source studies require a caveat that is not apparent from the pooled figures. In one [7], the score at final follow-up is the 12-month value in 78 hips, the same mean being reported at 24 months in only 55 hips; in another [29], the score derives from the 12 hips with complete paired measurements rather than the full cohort of 18. Both were retained with the sample size contributing to the paired measurement.

### 3.10. Retrieval Analyses

Three retrieval studies examined explanted constructs [8,17,18]. The consistent finding was minimal bone ingrowth into the tantalum. Apposition was largely confined to the lateral cortical entry point, with little or no bone within the portion of the construct sited in the necrotic segment; one quantitative morphometric analysis reported the ingrowth of approximately 10% at six weeks [17]. In the largest series, radiographic progression was present in 14 of 15 retrieved hips [8].

These findings are not amenable to formal appraisal, since retrieved implants are a selected sample of failures, and they cannot establish what proportion of implants fail to integrate. What they describe is the state of the construct in implants that did fail: apposition at the lateral cortical entry point, with little or no bone within the portion cited in the necrotic segment. The interpretation of that pattern is developed in the Section 4.

### 3.11. Conversion Arthroplasty After a Failed Implant

Three studies reported outcomes of arthroplasty performed after a failed rod [19,20,21], in 138 hips with follow-up from 64.1 to 88.8 months, each with a primary arthroplasty comparison group. Mean HHS after conversion was 91.9 [21], 95.2 [19], and 96.1 [20], from pre-conversion means of 56.7, 55.2, and 56.5, and all three reported no significant difference from primary arthroplasty in the outcomes assessed. Mean time from rod implantation to conversion was 33.1 months in the study reporting it [19]. Removal of the construct was described as requiring specific technique, with trephine or segmental removal to limit bone loss, and this consideration rather than the eventual functional result is the substantive caveat.

All three were retrospective studies with non-randomized comparison groups, and none was designed or powered to compare salvage arthroplasty with primary arthroplasty as a primary objective.

### 3.12. Certainty of Evidence

Certainty was rated very low for both primary outcomes (Table 3), starting at low for a body of evidence dominated by single-arm observational studies and downgraded further for inconsistency, given I-squared above 75% and prediction intervals spanning almost the full plausible range. The technique subgroup finding was rated very low, downgraded additionally for imprecision, for the dependence of statistical significance on a single internally controlled cohort, and for its dependence on the classification of three records whose procedural descriptions lie close to the boundary of that variable. The Harris Hip Score is presented descriptively and not as a pooled effect; the corresponding row of Table 3 records the certainty of that body of evidence rather than summarizing an estimate.

## 4. Discussion

Approximately one hip in five proceeds to arthroplasty after femoral head–preserving porous tantalum implantation, and radiographic deterioration occurs in approximately one in four. Both estimates carry prediction intervals so wide that the pooled values are weak guides to what any given series will report, and the central contribution of this review is an account of that dispersion rather than the pooled values themselves. Of the pre-specified explanations, only surgical technique was associated with outcome, and under the pre-specified classification it was associated with both primary endpoints in the same direction. That association is not robust: it loses statistical significance when any of three records near the boundary of the classification is moved across it, and it is reported here as the most plausible available account of the dispersion rather than as an established one. Neither geography, nor disease stage as codable here, nor implant generation, nor risk of bias, nor duration of follow-up accounted for the variation.

The pooled conversion estimate is consistent with the three previous syntheses despite drawing on roughly twice as many studies. Aurégan and colleagues reported conversion in 24.63% of 297 hips across seven studies at a mean of 26.97 months [14], and Onggo and colleagues reported that 77.9% of 550 hips remained radiographically stable over a mean of 3.1 years [15], while the earliest meta-analysis found an advantage for the rod over bone grafting on functional scores and collapse [13]. What the present analysis adds is not a different number but the separation of the two endpoints, which shows that radiographic progression is both more frequent than conversion and more variably reported. Progression exceeded conversion within the same cohort in twelve of sixteen records, and the gap reached fifty percentage points in one series. A synthesis that pools the two, or that reports whichever each source study happened to provide, will produce an estimate determined largely by the composition of its included studies. This is the most economical explanation for why the three earlier reviews are difficult to reconcile, and it is a reason to treat their apparent agreement or disagreement with caution rather than to adjudicate between them.

The absence of a detectable regional association runs against the prevailing impression, which arose from the chronological coincidence of declining reporting from North America and Europe and continued reporting from Asia [10]. Testing that impression formally shows no regional association, and the ordering of records by outcome does not follow national lines: the highest and lowest conversion proportions in this dataset each occur on both sides of that divide. Nor does device generation explain it. The only head-to-head randomized comparison of two tantalum devices, implanted by the same technique at the same center, found one conversion in 21 hips against none in 20, and the subgroup analysis across all records was likewise null. This matters because the introduction of newer wrought and additively manufactured devices is sometimes advanced as a reason to expect better contemporary results, and the available evidence does not support that expectation. Neither result demonstrates equivalence. With eight Western and fourteen Asian records, a single head-to-head device comparison of 41 hips and substantial residual heterogeneity, a moderate difference in either domain would not reliably have been detected; what these analyses support is that region and device generation do not account for the observed dispersion, not that outcomes are the same across regions or devices.

What did emerge is an association between outcome and whether a substrate for repair was supplied at the time of implantation. It is an association and not a demonstrated cause, for three reasons that are worth stating together: all but one of the comparisons are between the arms of different studies rather than within a randomized contrast; technique and geographical region are unevenly distributed across the same records, so the two are correlated even though region alone shows no association and does not survive adjustment; and the classification of technique is itself coarse enough that reclassifying three records removes the statistical significance of the contrast. Implantation of the rod alone was associated with conversion in 27.2% of hips against 13.9% where curettage and impaction grafting or biological augmentation were added, and with radiographic progression in 37.4% against 18.4%. The association survived adjustment for geographical region and accounted for approximately a quarter of between-study variance in the pre-specified coding, although not in every alternative coding examined. The most direct evidence is the single cohort in which the two techniques were compared internally, where conversion occurred in 21 of 44 hips after the rod alone against 7 of 50 with impaction grafting, and radiographic progression in 24 of 44 against 9 of 50. Concordance across two endpoints that are only partly correlated, in both between-study and within-study comparisons, is more informative than any of these results alone.

The clinical and retrieval evidence converge on a coherent mechanism for this. A porous tantalum construct provides immediate mechanical support to the subchondral plate, and no included series attributed failure to fracture or migration of the construct itself. Volumetric porosity from approximately 75% to 80% and an elastic modulus near 3 GPa, against roughly 2 GPa for subchondral and 1.2 GPa for cancellous bone, are close to those of the tissue it replaces [5], and in a canine transcortical model bone, it filled 13% of the pore volume at two weeks and from 42% to 53% at four weeks, with interface shear strength rising correspondingly [6]. What the construct does not do, in this indication, is integrate. All three retrieval series describe bone apposition confined largely to the lateral cortical entry point, with the intra-lesional portion remaining substantially unintegrated and one quantitative analysis reporting approximately 10% ingrowth at six weeks [8,17,18]. On the specimens available, the scaffold appears to act as a strut within necrotic bone rather than as a substrate for repair, so that the mechanical support it provides is not succeeded by biological fixation. Retrieved implants are a selected sample of failures and cannot establish how often integration occurs in implants that do not fail, so this is an account of the mode of failure and not of its frequency.

On that reading, the technique association is what would be predicted rather than an incidental finding. Implanting the rod alone leaves the necrotic segment in place and asks the construct to support it indefinitely; curettage with impaction grafting, or biological augmentation, replaces the substrate the construct must integrate with. The direction of the epidemiological signal matches the mechanism the histological studies describe, and the correspondence between two independent lines of evidence is stronger than either provides alone, though it remains coherence between two weak bodies of evidence rather than proof. A speculative corollary is that the decline in use may have been directed at the material when the difficulty lay in the indication. Porous tantalum osseointegrates in the vascularized host bed of an arthroplasty reconstruction, and the expectation that it would do so within a necrotic segment was an extrapolation that the retrieval specimens do not support. This is an interpretation built on two small and indirect bodies of evidence, and the present data do not establish it.

Three qualifications constrain how strongly the technique finding should be read, and the first is decisive. When the internally controlled cohort is removed, the between-group difference is no longer conventionally significant for either endpoint, and if both split publications are instead re-pooled as single records, it attenuates further. The aggregate signal depends materially on one study. Second, technique is unevenly distributed across regions, with seven of eight Western records using the rod alone against five of fourteen Asian records, so technique and region are confounded even though region alone shows no association and does not survive adjustment. Third, with the single exception noted, all comparisons are between study arms rather than randomized contrasts. Fourth, the classification itself is coarse and its boundary is not sharp: three of the twenty-two records describe procedures that could reasonably be placed on either side of it, and moving them singly or together removes the statistical significance of the contrast for both endpoints, leaving its direction intact for conversion but not for radiographic progression. Fifth, and least tractable, the choice of technique is not random: stage, lesion size and location, symptom severity and surgeon preference all plausibly influence it, and none could be adjusted for at record level, so confounding by indication cannot be excluded. The finding should be regarded as the most plausible available explanation for the dispersion and as a hypothesis for prospective testing, not as an established one.

How the primary result translates into harm depends on what failure costs the patient, and here, the salvage literature is reassuring as far as it goes. Across three studies comprising 138 hips, mean HHS after conversion arthroplasty was 91.9, 95.2, and 96.1, and none found a significant difference from primary arthroplasty in the outcomes assessed [19,20,21]. Removal of the construct was described as requiring trephine or segmental technique to limit bone loss, and that consideration rather than the eventual functional result is the substantive caveat. A conversion rate of one in five is therefore not equivalent to a harm rate of one in five: what the attempt at preservation costs, when it fails, is an interval of symptoms and a second operation rather than a compromised final result. This evidence rests on three retrospective studies and should not bear more weight than that.

For practice, these data do not support porous tantalum implantation as a stand-alone alternative to core decompression, which achieves a pooled success rate of approximately 65% in its own right and remains the reference procedure [4]. Where the tantalum procedure is undertaken, such evidence as exists is compatible with combining it with removal of necrotic bone and grafting, but that evidence is of very low certainty, depends substantially on one internally controlled cohort and is not by itself a sufficient basis for a recommendation. For research, the priority is not another single-arm series. The specific question raised by this synthesis, whether adding necrotic bone removal to rod implantation alters survivorship, is answerable by a randomized trial of modest size if the effect magnitude reported in that cohort is anywhere near correct. Such a trial should specify conversion to arthroplasty and radiographic progression as separate endpoints with blinded radiographic assessment, report survivorship at fixed time points, and record lesion size and location.

Several limitations qualify all the above. The evidence base is dominated by single-arm retrospective series at moderate or high risk of bias, and no analysis presented here can support causal inference; heterogeneity was substantial for both primary outcomes and only partly explained. Conversion to arthroplasty is not a protocol-defined event: no source study specified an indication for conversion, so the threshold at which a painful hip proceeds to arthroplasty varies with patient expectation, access to surgery, surgeon preference, and local practice, and some part of the dispersion in the conversion estimate reflects that variation rather than any difference in the treated disease. This is one reason the two endpoints were analyzed separately, since radiographic progression is less exposed to it, and it is also a reason for reporting the incidence analysis alongside the pooled proportion. Hips rather than patients are the unit of analysis, as in every source study; bilateral disease is common in this condition, two hips in one patient are not independent observations, and the intervals reported here are therefore likely to be somewhat narrower than the data warrant. Definitions of both endpoints varied between source studies: radiographic progression was assessed against three different classification systems, of which the ARCO classification was itself revised during the period covered [16], and conversion was reported in some studies alongside a broader clinical-failure category, the sensitivity analysis under which changed the pooled estimate by 1.1 percentage points. Restricting radiographic progression to one staging system at a time produced a significantly higher pooled proportion among the two studies using the Ficat classification, but those two records are also the smallest in the dataset and both used the rod alone, so staging system and technique cannot be separated there; the technique association itself persisted within the larger ARCO stratum. Disease stage could be modelled only at cohort level, and lesion size and location, plausibly the strongest patient-level predictors [3], were reported in a recognized classification by two records and three records, respectively, and not in the same classifications. Both bear directly on the mechanism proposed here, since ingrowth is expected at the interface with viable bone and therefore depends on how much viable bone the construct engages and on where within the lesion it lies; their near absence from this literature is the main reason that account cannot be tested against the clinical data and is offered as a hypothesis. Radiographic progression is a composite of stage advancement and femoral head collapse, which are not equivalent clinical events, and no source report separated the two, so the pooled value combines them in unknown and varying proportions. Conversion, clinical failure and radiographic progression are three different things, and the literature has not consistently kept them apart; one contribution of this review is to do so, but it can only do so at the level of what the source studies reported. The absence of an association between conversion and duration of follow-up should not be read as showing that follow-up duration is unimportant, since conversion is by construction cumulative; what the banded analyses and the incidence analysis show is that unequal observation time does not account for the dispersion between records, not that time is irrelevant to the individual patient. Etiology was not extractable in analyzable form, so the pre-specified subgroup analysis by etiology could not be performed; this is a deviation from the registered protocol and is reported as such, together with every other deviation, in Supplementary Table S1b. Functional outcome data were too heterogeneous to summarize meaningfully, with a prediction interval for the change in HHS spanning both deterioration and large improvement, and are reported descriptively rather than as a pooled effect. Restriction to English-language full texts may have excluded relevant reports, particularly from the Chinese literature where the procedure continues to be performed. The technique classification was applied by the review team from procedural descriptions of varying detail, and misclassification cannot be excluded. It is also a coarse variable. Among the records classified as using an adjunctive procedure, what was actually done ranges from partial curettage through the core track under endoscopic control [11] to complete excision of the necrotic segment through a window at the head–neck junction after surgical hip dislocation [12], and no study quantified the volume of necrotic bone removed or the volume remaining. Supplementary Table S3 sets out what each record reported. Because whether the necrotic segment was addressed is the principal explanation this review offers for the observed dispersion, the coarseness of that variable is a material limitation, and the finding identifies a direction for investigation rather than a graded relation between the extent of debridement and outcome. Two publications reporting distinct interventions separately were entered as two records apiece, which is defensible when pooling proportions since each record is an independent group of hips, but increases the weight of those cohorts in analyses counting records rather than patients; the technique finding is sensitive to that choice, and a sensitivity analysis re-pooling them is reported alongside the primary estimate. Finally, the salvage and retrieval domains rest on three studies each and inform interpretation rather than support estimation.

## 5. Conclusions

Femoral head–preserving porous tantalum implantation is followed by conversion to arthroplasty in approximately one hip in five and by radiographic deterioration in approximately one in four, with dispersion between records so wide that the pooled values are of limited predictive use. Geography, disease stage, implant generation, and follow-up duration do not explain that dispersion; whether a substrate for repair was supplied at implantation accounted for part of the between-study variation, on both endpoints and in the same direction, although the size and the significance of that association depend on how three records near the boundary of that classification are coded, and retrieval evidence indicates that the construct does not osseointegrate within necrotic bone. That the limiting factor is the biological substrate rather than the material is a hypothesis supported by convergent but indirect evidence rather than a demonstrated mechanism. These data do not support use of the device as a stand-alone alternative to core decompression, and the question of whether combining it with debridement and grafting alters its performance is well defined, clinically consequential, and answerable by a trial that has not yet been done.

## Figures and Tables

**Figure 1:**
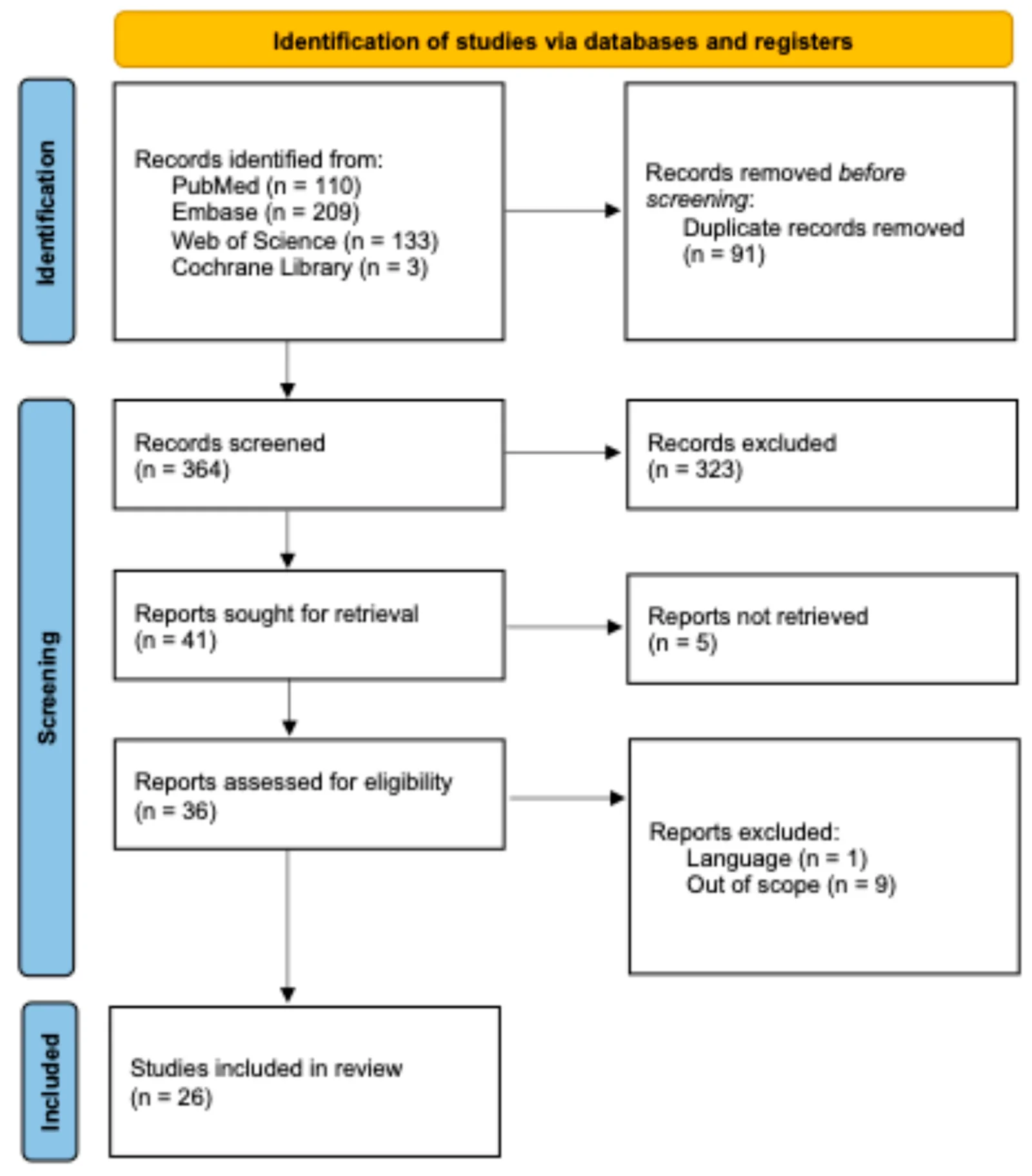
Flow diagram of the literature search and studies selection [22].

**Figure 2:**
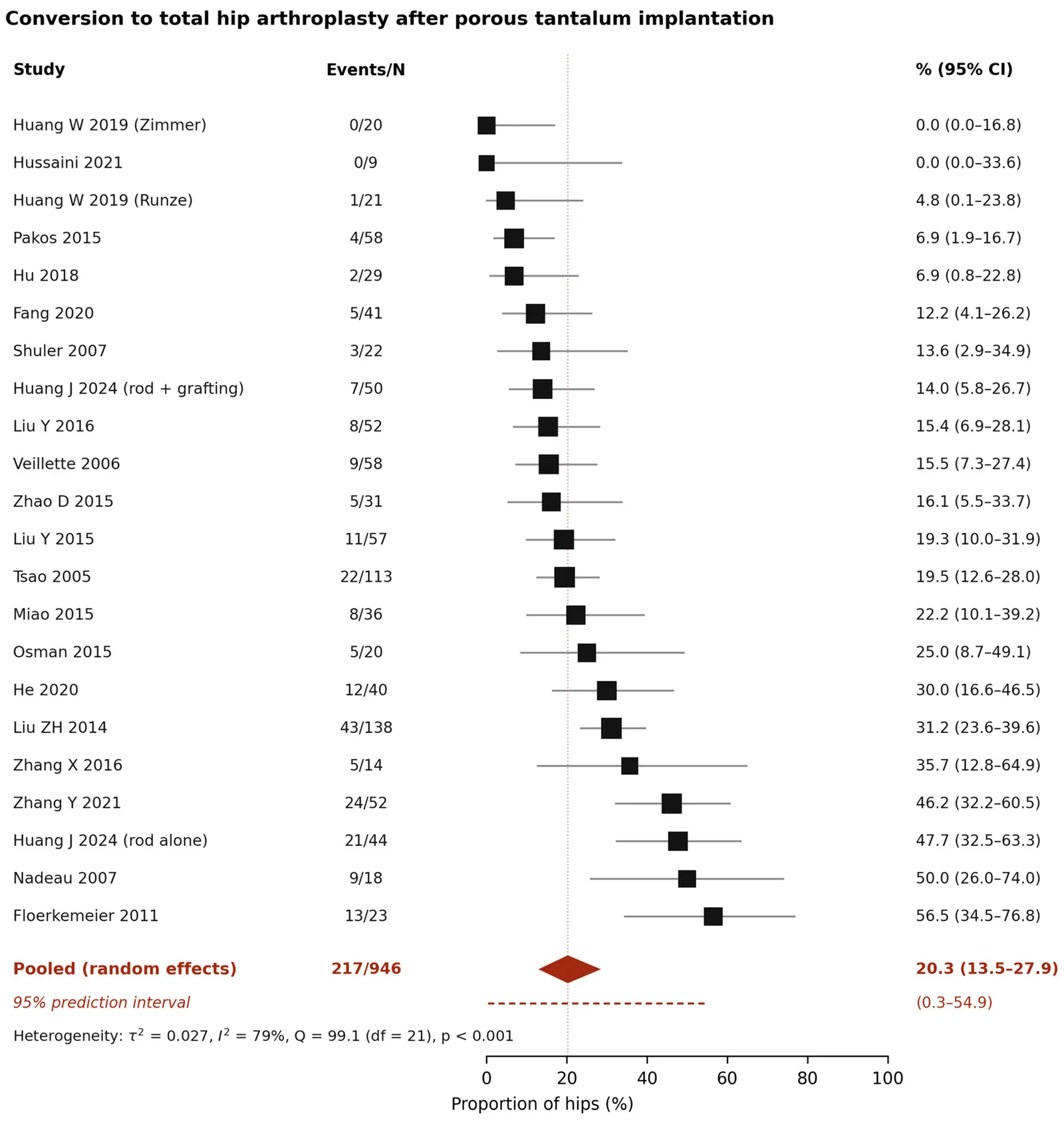
Forest plot of conversion to total hip arthroplasty after femoral head–preserving porous tantalum implantation. Marker size is proportional to random-effects weight; horizontal lines are exact binomial 95% confidence intervals. The diamond is the pooled random-effects estimate, and the dashed line is the 95% prediction interval [7,9,11,12,18,26,28,29,30,31,32,33,34,35,36,37,38,39,40,41].

**Figure 3:**
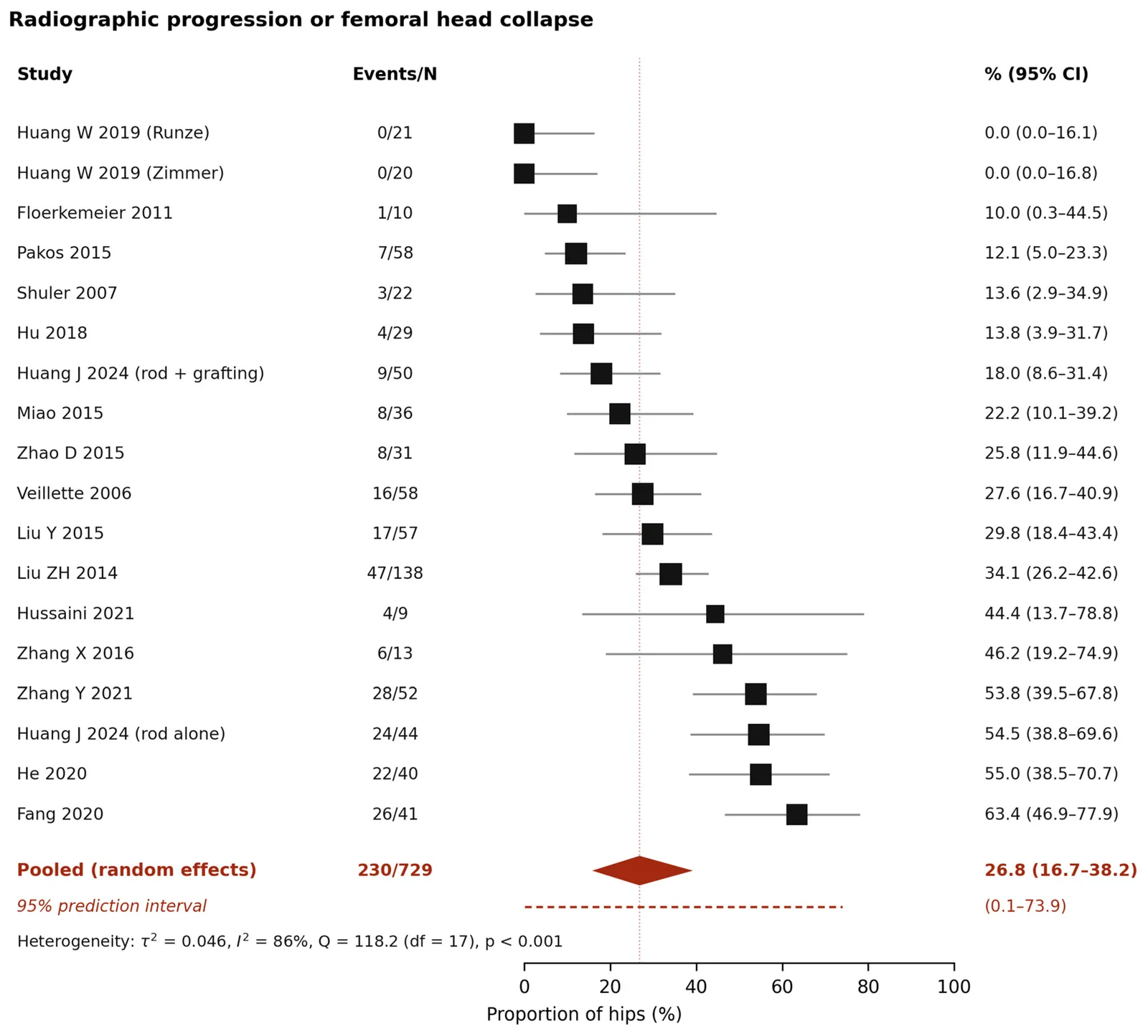
Forest plot of radiographic progression or femoral head collapse. Definitions follow the classification system used by each source study; the system and the endpoint applied by each record are given in Supplementary Table S2 [9,11,12,18,26,28,30,31,32,33,35,37,38,39,40,41].

**Figure 4:**
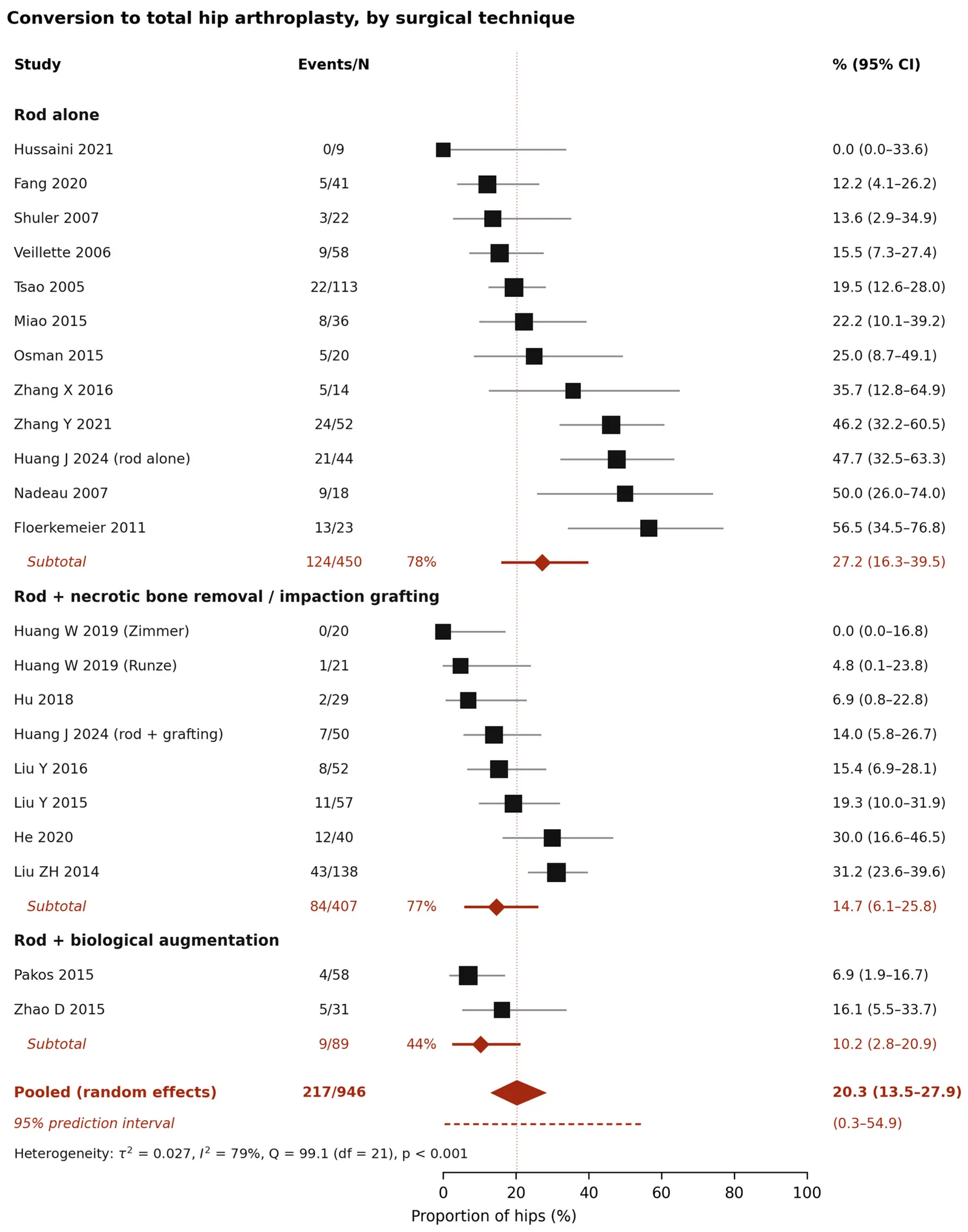
Conversion to total hip arthroplasty stratified by surgical technique. The two publications reporting distinct interventions separately are entered as two records apiece, so that no cohort of hips contributes twice [7,9,11,12,18,26,28,29,30,31,32,33,34,35,36,37,38,39,40,41].

**Figure 5:**
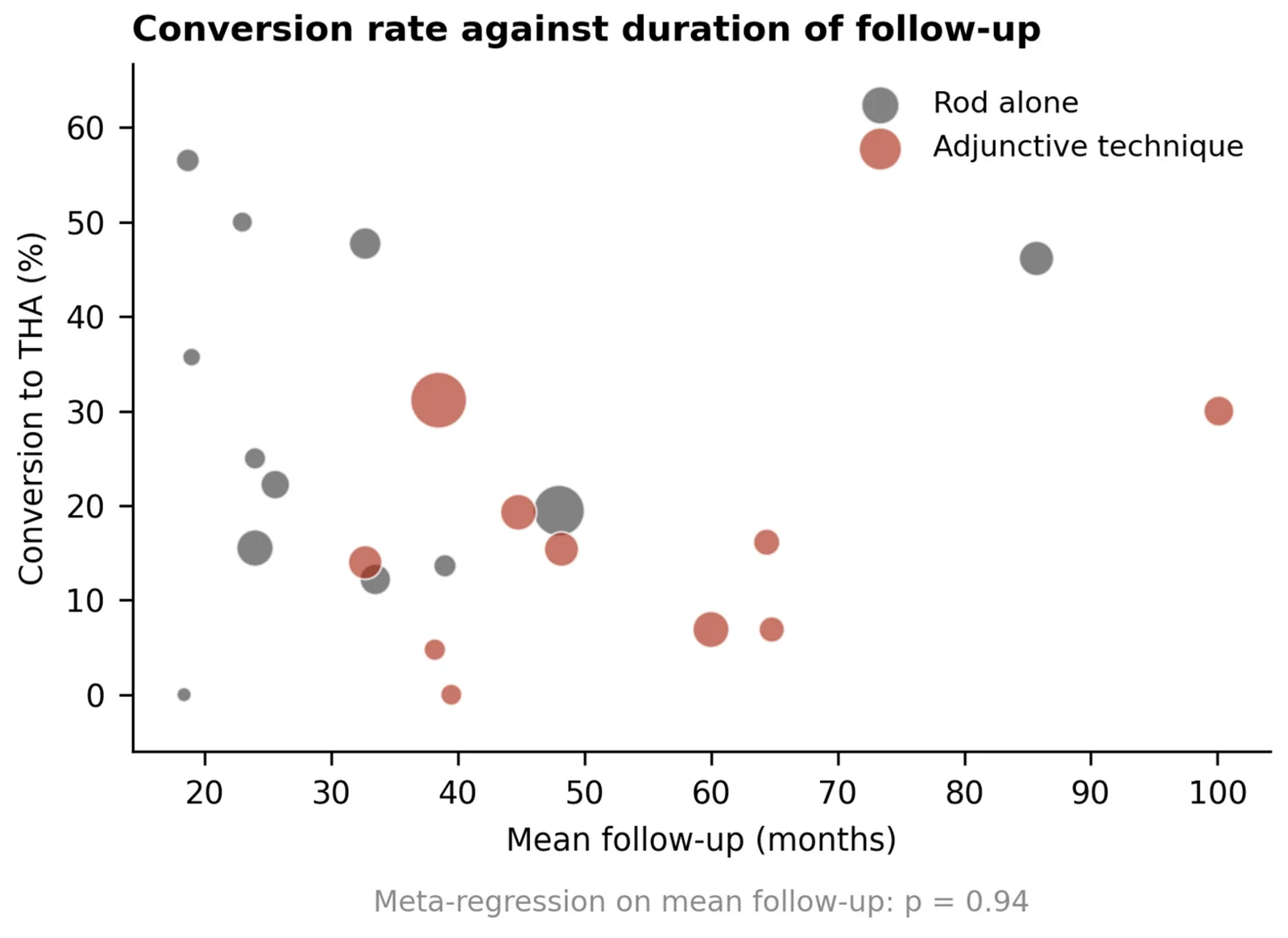
Conversion rate against mean duration of follow-up, with marker area proportional to cohort size and color denoting surgical technique. No association was found on meta-regression.

**Table 3:** GRADE summary of findings.

Outcome	Records (Hips)	Effect	Downgraded for	GRADE
Conversion to total hip arthroplasty	22 (946)	20.3% (95% CI 13.5–27.9)	Study design (observational, single-arm); inconsistency (I^2^ 78.8%, prediction interval 0.3–54.9%)	Very low
Radiographic progression or collapse	18 (729)	26.8% (95% CI 16.7–38.2)	Study design; inconsistency (I^2^ 85.6%); indirectness (three staging systems, assessor-determined endpoint, no blinding)	Very low
Effect of adjunctive technique on conversion	22 (946)	27.2% vs. 13.9% (*p* = 0.028)	Study design (non-randomized between-arm comparison); imprecision; significance dependent on one cohort and on the classification of three records	Very low
Effect of adjunctive technique on radiographic progression	18 (729)	37.4% vs. 18.4% (*p* = 0.041)	As above, with additional imprecision; significance not retained under any alternative classification examined	Very low
Harris Hip Score, change from baseline	9 (302)	Not pooled; reported descriptively	Study design; inconsistency (I^2^ 99%, prediction interval −14.6 to 53.4); no control arms	Very low
Outcome of conversion arthroplasty after failure	3 (138)	No significant difference from primary arthroplasty	Study design (retrospective); imprecision; small number of studies	Very low

All outcomes start at low certainty as a body of evidence dominated by single-arm observational studies. No outcome was upgraded.

## Data Availability

No new data were created or analyzed in this study. Data sharing is not applicable to this article.

## Supplementary Materials

The following supporting information can be downloaded at: https://www.mdpi.com/article/10.3390/medsci14060640/s1. The extracted dataset, the data-extraction form and the analysis code are available from the corresponding author on reasonable request. Full search strategies are provided in Supplementary File S1, the registration and amendment history and the deviations from the registered protocol in Supplementary Table S1a,b, and the definition of radiographic progression applied by each source study in Supplementary Table S2, and the handling of the necrotic segment, the material placed in the femoral head and the reporting of lesion size and location in Supplementary Table S3.
