# Supplementary material for: Femoral Head–Preserving Porous Tantalum Implantation for Osteonecrosis of the Femoral Head: A Systematic Review and Meta-Analysis of Conversion to Total Hip Arthroplasty, Radiographic Progression, and Modes of Failure
Source: Med Sci (Basel). 2026 Oct 5;14(6):640. doi: 10.3390/medsci14060640 (PMC13707016; doi:10.3390/medsci14060640)
Supplement: Supplementary file 1 [file medsci-14-00640-s001.zip › medsci-4583972 PRISMA_2020_checklist_AVN_tantalum_completed.pdf]

PRISMA 2020 Checklist

Section and Topic	Item #	Checklist item	Location where item is reported
**TITLE**			
Title	1	Identify the report as a systematic review.	Title (page 1): identified as “a systematic review and meta-analysis”.
**ABSTRACT**			
Abstract	2	See the PRISMA 2020 for Abstracts checklist.	Abstract (page 1), structured as Background / Methods / Results / Conclusions. The PROSPERO registration number is not given in the Abstract (PRISMA for Abstracts item 12).
**INTRODUCTION**			
Rationale	3	Describe the rationale for the review in the context of existing knowledge.	Section 1 (Introduction), paragraphs 1–4.
Objectives	4	Provide an explicit statement of the objective(s) or question(s) the review addresses.	Section 1 (Introduction), final paragraph.
**METHODS**			
Eligibility criteria	5	Specify the inclusion and exclusion criteria for the review and how studies were grouped for the syntheses.	Section 2.2 (Eligibility criteria). Grouping of studies for the syntheses: Sections 2.5 and 2.7, and Table 1.
Information sources	6	Specify all databases, registers, websites, organisations, reference lists and other sources searched or consulted to identify studies. Specify the date when each source was last searched or consulted.	Section 2.3 (Information sources and search): MEDLINE (PubMed), Embase, Web of Science and the Cochrane Library, supplemented by hand-searching reference lists of included articles and of previous systematic reviews; searched to September 2025. The exact date on which each source was last searched is not given (month only).
Search strategy	7	Present the full search strategies for all databases, registers and websites, including any filters and limits used.	Section 2.3 and Supplementary File 1 (full search strategies for each database).
Selection process	8	Specify the methods used to decide whether a study met the inclusion criteria of the review, including how many reviewers screened each record and each report retrieved, whether they worked independently, and if applicable, details of automation tools used in the process.	Section 2.4 (Study selection and data extraction).
Data collection process	9	Specify the methods used to collect data from reports, including how many reviewers collected data from each report, whether they worked independently, any processes for obtaining or confirming data from study investigators, and if applicable, details of automation tools used in the process.	Section 2.4 (standardized extraction form; variables extracted; internal discrepancies resolved against the full text).
Data items	10a	List and define all outcomes for which data were sought. Specify whether all results that were compatible with each outcome domain in each study were sought (e.g. for all measures, time points, analyses), and if not, the methods used to decide which results to collect.	Section 2.5 (Outcomes): co-primary outcomes (conversion to THA at longest available follow-up; radiographic progression as defined by the source study) and secondary outcomes.
	10b	List and define all other variables for which data were sought (e.g. participant and intervention characteristics, funding sources). Describe any assumptions made about any missing or unclear information.	Section 2.4 (list of extracted study-, patient-, implant- and technique-level variables; handling of internal discrepancies). Assumptions about missing or unclear information are described where relevant in

Section and Topic	Item #	Checklist item	Location where item is reported
			Sections 2.7, 3.1 and 3.8.
Study risk of bias assessment	11	Specify the methods used to assess risk of bias in the included studies, including details of the tool(s) used, how many reviewers assessed each study and whether they worked independently, and if applicable, details of automation tools used in the process.	Section 2.6 (Risk of bias): RoB 2 for randomized trials, ROBINS-I for non-randomized comparative studies, MINORS for single-arm cohorts and case series; retrieval analyses appraised narratively. Not reported: how many reviewers assessed each study and whether they worked independently.
Effect measures	12	Specify for each outcome the effect measure(s) (e.g. risk ratio, mean difference) used in the synthesis or presentation of results.	Section 2.7 (Synthesis): proportions (Freeman–Tukey double arcsine transformation) for both primary outcomes; risk ratio and risk difference for the internally controlled comparison (Section 3.5); raw mean change and standardized mean change for the Harris Hip Score.
Synthesis methods	13a	Describe the processes used to decide which studies were eligible for each synthesis (e.g. tabulating the study intervention characteristics and comparing against the planned groups for each synthesis (item #5)).	Sections 2.2, 2.5 and 2.7; Section 3.1 and Table 1 (allocation of records to the efficacy, retrieval and salvage domains; handling of the two publications reporting two interventions separately).
	13b	Describe any methods required to prepare the data for presentation or synthesis, such as handling of missing summary statistics, or data conversions.	Section 2.7 (Freeman–Tukey transformation with back-transformation at the harmonic mean sample size; exact binomial CIs for individual studies; pre–post correlation imputed at 0.5 for the HHS; studies reporting medians and interquartile ranges excluded rather than converted).
	13c	Describe any methods used to tabulate or visually display results of individual studies and syntheses.	Section 2.7; Tables 1–3 and Figures 2–6 (forest plots, meta-regression scatter plot, funnel plot).
	13d	Describe any methods used to synthesize results and provide a rationale for the choice(s). If meta-analysis was performed, describe the model(s), method(s) to identify the presence and extent of statistical heterogeneity, and software package(s) used.	Section 2.7: random-effects model, REML estimation of between-study variance, Hartung–Knapp adjusted confidence intervals, τ^2, I^2 and Cochran's Q with 95% prediction intervals; analyses performed in Python 3 using purpose-written implementations.
	13e	Describe any methods used to explore possible causes of heterogeneity among study results (e.g. subgroup analysis, meta-regression).	Section 2.7: pre-specified subgroup analyses (surgical technique, disease stage, region, implant generation, study design, risk of bias) tested by Q between groups, and meta-regression including a bivariable model with region held constant.

PRISMA 2020 Checklist

Section and Topic	Item #	Checklist item	Location where item is reported
	13f	Describe any sensitivity analyses conducted to assess robustness of the synthesized results.	Section 2.7: leave-one-out estimation, exclusion of studies at high risk of bias, exclusion of the pediatric cohort, restriction to mean follow-up ≥ 24 months, and re-analysis under the broader clinical-failure definition.
Reporting bias assessment	14	Describe any methods used to assess risk of bias due to missing results in a synthesis (arising from reporting biases).	Section 2.7 (funnel plot and Egger's regression test for small-study effects).
Certainty assessment	15	Describe any methods used to assess certainty (or confidence) in the body of evidence for an outcome.	Section 2.7 (GRADE, with a starting level of low for a body of evidence dominated by single-arm observational studies).
**RESULTS**			
Study selection	16a	Describe the results of the search and selection process, from the number of records identified in the search to the number of studies included in the review, ideally using a flow diagram.	Section 3.1 and Figure 1 (PRISMA 2020 flow diagram).
	16b	Cite studies that might appear to meet the inclusion criteria, but which were excluded, and explain why they were excluded.	Section 3.1 and Table 1
Study characteristics	17	Cite each included study and present its characteristics.	Table 1 and Section 3.1 (region, design, hips, follow-up, stage, technique, device and events for each included record).
Risk of bias in studies	18	Present assessments of risk of bias for each included study.	Section 3.2 and the risk-of-bias column of Table 1.
Results of individual studies	19	For all outcomes, present, for each study: (a) summary statistics for each group (where appropriate) and (b) an effect estimate and its precision (e.g. confidence/credible interval), ideally using structured tables or plots.	Table 1 (events/hips for both primary outcomes in each record) and Figures 2 and 3 (per-study proportions with exact binomial 95% confidence intervals).
Results of syntheses	20a	For each synthesis, briefly summarise the characteristics and risk of bias among contributing studies.	Sections 3.1 and 3.2 (characteristics and risk of bias of the contributing records) and the opening paragraphs of Sections 3.3–3.5.
	20b	Present results of all statistical syntheses conducted. If meta-analysis was done, present for each the summary estimate and its precision (e.g. confidence/credible interval) and measures of statistical heterogeneity. If comparing groups, describe the direction of the effect.	Sections 3.3, 3.4, 3.5 and 3.8; Figures 2–4; Tables 2 and 3 (pooled estimates with 95% CIs, τ^2, I^2, Q and 95% prediction intervals).
	20c	Present results of all investigations of possible causes of heterogeneity among study results.	Sections 3.5 and 3.6 (subgroup analyses by technique, stage, region, implant generation, design and risk of bias; meta-regression, including the bivariable model).
	20d	Present results of all sensitivity analyses conducted to assess the robustness of the synthesized results.	Section 3.7 and Table 2 (all pre-specified sensitivity analyses, including leave-one-out estimates).
Reporting biases	21	Present assessments of risk of bias due to missing results (arising from reporting biases) for each synthesis assessed.	Section 3.7 and Figure 6 (funnel plot and Egger's regression test).

PRISMA 2020 Checklist

Section and Topic	Item #	Checklist item	Location where item is reported
Certainty of evidence	22	Present assessments of certainty (or confidence) in the body of evidence for each outcome assessed.	Section 3.11 and Table 3 (GRADE summary of findings for all six outcomes).
**DISCUSSION**			
Discussion	23a	Provide a general interpretation of the results in the context of other evidence.	Section 4 (Discussion), paragraphs 1–7.
	23b	Discuss any limitations of the evidence included in the review.	Section 4, final paragraph (risk of bias of the evidence base, heterogeneity, varying endpoint definitions and staging systems, cohort-level stage coding, unreported lesion size and location, non-extractable etiology).
	23c	Discuss any limitations of the review processes used.	Section 4, final paragraph (restriction to English-language full texts, technique classification applied by the review team, entry of two publications as two records apiece and its influence on the technique finding, and the protocol deviation regarding the etiology subgroup).
	23d	Discuss implications of the results for practice, policy, and future research.	Section 4 (paragraph beginning “For practice…”) and Section 5 (Conclusions).
**OTHER INFORMATION**			
Registration and protocol	24a	Provide registration information for the review, including register name and registration number, or state that the review was not registered.	Section 2.1 (Protocol and registration): PROSPERO, CRD420251151228.
	24b	Indicate where the review protocol can be accessed, or state that a protocol was not prepared.	Section 2.1: the review was conducted according to the registered PROSPERO protocol; a direct link to the record is not given.
	24c	Describe and explain any amendments to information provided at registration or in the protocol.	Section 2.1 (amendment recorded before analysis, dividing the registered program of work into separate reviews by clinical problem) and Section 4, final paragraph (subgroup analysis by etiology not performed, reported as a deviation from the registered protocol).
Support	25	Describe sources of financial or non-financial support for the review, and the role of the funders or sponsors in the review.	Funding statement (back matter): no external funding.
Competing interests	26	Declare any competing interests of review authors.	Conflicts of Interest statement (back matter): the authors declare no conflicts of interest.
Availability of data, code and other materials	27	Report which of the following are publicly available and where they can be found: template data collection forms; data extracted from included studies; data used for all analyses; analytic code; any other materials used in the review.	Data Availability Statement (back matter)

From: Page MJ, McKenzie JE, Bossuyt PM, Boutron I, Hoffmann TC, Mulrow CD, et al. The PRISMA 2020 statement: an updated guideline for reporting systematic reviews. BMJ 2021;372:n71. doi:

PRISMA 2020 Checklist

10.1136/bmj.n71. This work is licensed under CC BY 4.0. To view a copy of this license, visit https://creativecommons.org/licenses/by/4.0/
