# Supplementary material for: Femoral Head–Preserving Porous Tantalum Implantation for Osteonecrosis of the Femoral Head: A Systematic Review and Meta-Analysis of Conversion to Total Hip Arthroplasty, Radiographic Progression, and Modes of Failure
Source: Med Sci (Basel). 2026 Oct 5;14(6):640. doi: 10.3390/medsci14060640 (PMC13707016; doi:10.3390/medsci14060640)
Supplement: Supplementary file 1 [file medsci-14-00640-s001.zip › medsci-4583972-supplementary.pdf]

Supplementary Material

Femoral head–preserving porous tantalum implantation for osteonecrosis of the femoral head: a systematic review and meta-analysis of conversion to total hip arthroplasty, radiographic progression, and modes of failure

Supplementary Table S1a. Registration, search, amendment and analysis history of the review.

Stage	Date	What was registered or done	Relation to the present review
PROSPERO registration submitted	19 September 2025	Record CRD420251151228, "Tantalum as a Model for Porous Metals in Orthopedic Reconstruction". Population, intervention, comparators, outcomes, sources, eligibility criteria, risk-of-bias instruments and synthesis plan registered; a full protocol was uploaded to PROSPERO.	Inception record for the programme of work of which this review is one part.
Registration published (version 1.0)	21 September 2025	Public record available at the registration link.	—
Search executed	to 19 September 2025	The registered strategy combines a tantalum concept block with a broad indication block: (tantalum OR trabecular metal OR porous tantalum) AND (hip OR knee OR shoulder OR ankle OR foot OR arthroplasty OR revision OR cone OR augment), translated into the syntax and controlled vocabulary of each database. T	To narrow the retrieved results, the search was restricted to studies concerning osteonecrosis of the femoral head.
Screening completed	September 2025 to July 2026	Titles, abstracts and full texts screened in duplicate against the registered eligibility criteria across all tantalum indications. 181 reports met those criteria.	One screening process and one extraction dataset serve all four reviews.
Amendment published (version 1.1)	6 August 2026	The eligible literature was divided into four reviews by clinical problem: fixation in intact host bone; reconstruction of bone loss; femoral head–preserving surgery in osteonecrosis; and low-volume and emerging indications. Each report is allocated to one review, each review reports its own PRISMA flow diagram derived from the common search, and the division is by indication rather than by outcome.	Defines the present review. The amendment states that the search, the eligibility criteria and the outcome definitions registered at inception are unchanged; the outcome deviation recorded in the second panel below is an exception to that statement and is declared here.
Analysis of the present review	September 2026	Pre-specified synthesis: Freeman–Tukey random-effects pooling of the two primary outcomes separately, pre-specified subgroup analyses and meta-regression, pre-specified sensitivity analyses, GRADE.	Carried out after the amendment was published, not before it.
Analyses added after peer review	September 2026	Sensitivity analysis of radiographic progression by staging system with the technique comparison repeated within the ARCO stratum; estimation of conversion per hip-year and within bands of comparable follow-up; restriction of the paired comparison to records with matched denominators; removal in turn of two possibly overlapping reports; progressive raising of the minimum cohort size; reclassification of three records near the boundary of the technique variable; variation of the imputed pre–post correlation for the Harris Hip Score.	Identified as post hoc wherever reported. Neither primary estimate changed.

The registration covers a programme of work examining porous tantalum across orthopaedic indications. One search, one screening process and one extraction dataset serve all four reviews arising from it. The amendment divided that programme by clinical problem and did not alter the search or the eligibility criteria. The review is therefore prospectively registered with respect to its search and its eligibility criteria, and retrospectively amended with respect to its scope and, in one respect recorded below, its outcomes.

Supplementary Table S1b. Deviations from the registered protocol.

Registered at inception	What was done in this review	Reason	Declared in
Case series with at least 10 patients eligible; case reports with fewer than 10 patients excluded.	Reports of at least five hips eligible.	Hips rather than patients are the unit of analysis throughout this literature, and the two thresholds are not interchangeable in a condition that is frequently bilateral. Two included reports fall between them.	Section 2.2; sensitivity analysis in Section 3.7 raising the threshold to 10, 15 and 20 hips (20.3% at base case, 21.2%, 20.7% and 19.7% respectively).
MEDLINE, PubMed, Science Citation Index, Social Sciences Citation Index, Scopus and Web of Science.	MEDLINE (PubMed), Embase, Web of Science and the Cochrane Library (CENTRAL); Scopus used for forward citation tracking rather than as a primary source.	Embase and CENTRAL were added because neither was covered by the registered set and both index orthopaedic reports and trials that MEDLINE does not.	Section 2.3 and Supplementary File 1.
Databases searched for articles published from 1 January 1997.	No date limit applied at the search stage.	The registered limit would not have excluded any eligible report, the earliest included report dating from 2005, and an arbitrary truncation was avoided.	Section 2.3.
Main outcome: revision of the index tantalum implant for any cause. Additional outcomes: revision for aseptic loosening; revision for infection; dislocation; union; functional scores; other complications; blood tantalum; evidence of ALVAL.	Co-primary outcomes: conversion to total hip arthroplasty, and radiographic progression. Both analysed separately and never pooled.	Conversion to arthroplasty is the registered main outcome under the name it carries in this indication. Radiographic progression was not among the registered outcomes and was raised to co-primary status at review level, before any analysis, because the two events occur at different frequencies in the same hips and previous syntheses had conflated them. This is the substantive deviation from the registration and is declared as such.	Section 2.5 and this table. A further amendment recording the review-specific outcome set has been requested.
Funnel plots and Egger's regression test when at least 10 studies are available; trim-and-fill as a sensitivity check.	Egger's test applied to the two primary pooled estimates and to no subgroup. Trim-and-fill not performed.	The registered threshold of ten studies was applied as registered. Trim-and-fill was not performed because no estimate showed funnel-plot asymmetry and no subgroup reached ten records.	Section 2.7 and Section 3.7.
Subgroup analysis by etiology of osteonecrosis.	Not performed.	Etiology was not reported in extractable form by a sufficient number of studies.	Section 4.

Deviations are listed in full, including those that did not affect any estimate. The fourth entry is the one that bears on the interpretation of the review: radiographic progression was not a registered outcome, and its elevation to co-primary status is the central methodological decision of this paper. It was taken at review level before any analysis was run, it is

the reason the review exists in its present form, and it is declared here rather than left to be discovered from the public record.

Supplementary Table S2. Radiographic staging system and progression endpoint applied in each contributing record.

Record	Staging system	Stage at implantation	Progression endpoint as applied	n/N
Floerkemeier 2011 [30]	ARCO	ARCO I–II	Advancement of at least one ARCO stage, or femoral head collapse	1/10
Liu ZH 2014 [31]	ARCO	ARCO II–III	Advancement of at least one ARCO stage, or femoral head collapse	47/138
Zhao D 2015 [35]	ARCO	ARCO IIIc–IV	Advancement of at least one ARCO stage, or femoral head collapse	8/31
Zhang X 2016 [18]	ARCO	ARCO II–III	Advancement of at least one ARCO stage, or femoral head collapse	6/13
Hu 2018 [37]	ARCO	ARCO I–II	Advancement of at least one ARCO stage, or femoral head collapse	4/29
Huang W 2019 (Runze) [26]	ARCO	ARCO I–IIB	Advancement of at least one ARCO stage, or femoral head collapse	0/21
Huang W 2019 (Zimmer) [26]	ARCO	ARCO I–IIB	Advancement of at least one ARCO stage, or femoral head collapse	0/20
He 2020 [39]	ARCO	ARCO II–III	Advancement of at least one ARCO stage, or femoral head collapse	22/40
Zhang Y 2021 [40]	ARCO	ARCO I–II	Advancement of at least one ARCO stage, or femoral head collapse	28/52
Huang J 2024 (rod alone) [12]	ARCO	ARCO IIa–IIc	Advancement of at least one ARCO stage, or femoral head collapse	24/44
Huang J 2024 (rod + graft) [12]	ARCO	ARCO IIa–IIc	Advancement of at least one ARCO stage, or femoral head collapse	9/50
Veillette 2006 [28]	Steinberg	Steinberg I–III	Advancement of at least one Steinberg stage, or femoral head collapse	16/58
Liu Y 2015 [32]	Steinberg	Steinberg I–IVA	Advancement of at least one Steinberg stage, or femoral head collapse	17/57
Miao 2015 [33]	Steinberg	Steinberg I–II	Advancement of at least one Steinberg stage, or femoral head collapse	8/36
Pakos 2015 [11]	Steinberg	Steinberg II–IIIa	Advancement of at least one Steinberg stage, or femoral head collapse	7/58
Fang 2020 [38]	Ficat	Ficat I–II	Advancement of at least one Ficat stage, or femoral head collapse	26/41
Hussaini 2021 [41]	Ficat	Ficat I–III	Advancement of at least one Ficat stage, or femoral head collapse	4/9
Shuler 2007 [9]	Univ. of Pennsylvania	UPenn I–II	Advancement of at least one University of Pennsylvania stage, or femoral head collapse	3/22

Records are ordered by staging system and then by year. n/N is the number of hips with radiographic progression over the number of hips in which progression was assessed; this denominator is smaller than the cohort size in two records in which radiographic follow-up was incomplete. Where a source report did not state a numerical threshold for progression, the review definition was applied during extraction, namely advancement of at least one grade in the

classification used by that report, or femoral head collapse as defined by that report. The ARCO classification was revised in 2019, so records published before and after that year are not strictly commensurable even within the ARCO stratum.

Pooled proportions by staging system (post hoc sensitivity analysis, Section 3.4): ARCO 25.4% (95% CI 10.8–43.1; k = 11, 149/448 hips; I^2 88.0%); Steinberg 22.5% (95% CI 10.5–37.3; k = 4, 48/209; I^2 54.5%); Ficat 60.2% (95% CI 45.1–74.5; k = 2, 30/50; I^2 3.3%); University of Pennsylvania not pooled (k = 1, 3/22). Q between groups = 19.58, df = 2, $p < 0.001$. Within the ARCO stratum, rod alone 44.1% (95% CI 14.8–75.7; k = 4) against an adjunctive procedure 17.7% (95% CI 2.8–40.2; k = 7); Q between groups = 4.13, df = 1, p = 0.042.

Supplementary Table S3. Handling of the necrotic segment, material placed in the femoral head, and reporting of lesion size and location, by record.

Record	Handling of the necrotic segment as reported	Material placed in the femoral head	Lesion size / location	Class
Tsao 2005 [7]	Core-decompression tract prepared and the rod advanced to abut the subchondral plate; no debridement of the lesion described.	None	NR / NR	Rod alone
Veillette 2006 [28]	Core decompression with 8, 9 and 10 mm cannulated reamers to subchondral level; no debridement beyond the reamed tract.	None	NR / NR	Rod alone
Nadeau 2007 [29]	A 10 mm core channel reamed over a guide pin and the plug inserted; no debridement described.	None	NR / NR	Rod alone
Shuler 2007 [9]	Implanted according to the manufacturer's surgical technique; no debridement described.	None	NR / NR	Rod alone
Floerkemeier 2011 [30]	Progressive reaming to 10 mm with the guide-pin tip about 5 mm from the endosteal surface; no debridement described.	None	NR / NR	Rod alone
Liu ZH 2014 [31]	Necrotic tissue curetted to subchondral bone in some patients; the proportion in whom this was done is not stated.	Autologous iliac bone with 5 g demineralized bone matrix and BMP-rich DBM, layered into the cavity	Large 65, medium 64, small 39 / lateral 63, central 68, medial 35	Rod + graft
Liu Y 2015 [32]	Decompression to subchondral level with 8, 9 and 10 mm cannulated drills; necrotic bone and sequestrum removed with a long curette and expanding scrapers.	Granular nano-hydroxyapatite / polyamide-66 composite, filled and compacted repeatedly	NR / NR	Rod + graft
Miao 2015 [33]	Core reamed to 10 mm over a guide pin and the rod threaded into position; no debridement described.	None	NR / NR	Rod alone
Osman 2015 [34]	Tunnel reamed to 10 mm with the rod tip within 5 mm of the articular surface; no debridement described.	None	NR / NR	Rod alone
Pakos 2015 [11]	Endoscopic inspection through the canal with partial curettage of the lesion using a threaded flexible Kirschner wire.	Morselized reaming autograft with iliac-crest marrow aspirate; rod soaked in aspirate for 5 min	NR / NR	Rod + biologic
Zhao D 2015 [35]	Necrotic lesion excised through a 3 × 2 cm window at the anterior head–neck junction.	Cultured autologous marrow stromal cells with cancellous iliac chips, plus a pedicled vascularized iliac graft	NR / NR	Rod + biologic

Record	Handling of the necrotic segment as reported	Material placed in the femoral head	Lesion size / location	Class
Liu Y 2016 [36]	Wide excision of necrotic bone, by the modified technique of the authors' earlier report.	Porous composite bone-filling material	≥ 30% of the head in 21/52 hips / NR	Rod + graft
Zhang X 2016 [18]	Reaming over a guide pin to the center of the lesion; no debridement described.	None	NR / JIC A, C1 or C2	Rod alone
Hu 2018 [37]	Decompression and clearance of the lesion with a dedicated drill under real-time CT guidance.	Not stated	NR / NR	Rod + graft [a]
Huang W 2019 (Runze) [26]	Core reaming to 8–10 mm; necrotic bone described as removed thoroughly.	Autologous bone chips placed in the excavated region before rod insertion	NR / NR	Rod + graft
Huang W 2019 (Zimmer) [26]	Core reaming to 8–10 mm; necrotic bone described as removed thoroughly.	Autologous bone chips placed in the excavated region before rod insertion	NR / NR	Rod + graft
Fang 2020 [38]	Tunnel enlarged to 10 mm; osteonecrotic bone removed with a curette and the tunnel irrigated.	None	NR / NR	Rod alone [b]
He 2020 [39]	Core decompression through a 10 mm tunnel; allograft placed in the head and impacted to support it.	Impacted allograft	NR / JIC C1 in 30 hips, C2 in 10	Rod + graft
Zhang Y 2021 [40]	A 10 mm core reamer used; necrotic tissue and the surrounding sclerotic area scraped out.	None	NR / NR	Rod alone [b]
Hussaini 2021 [41]	Entry point chosen proximal to the lesser trochanter to reduce fracture risk; no debridement described.	None	NR / NR	Rod alone
Huang J 2024 (rod alone) [12]	Rod implanted through the core track without opening the joint; no debridement described.	None	NR / NR	Rod alone
Huang J 2024 (rod + graft) [12]	Surgical hip dislocation, window at the head–neck junction, thorough removal of necrotic tissue under direct vision.	Cancellous autograft from the greater trochanter or the contralateral femoral head, or allograft, impaction grafted	NR / NR	Rod + graft

NR, not reported. Records are ordered as in Table 1 of the main text. The entries reproduce what each source report describes; no study quantified the volume of necrotic bone removed or the volume remaining, and none reported the proportion of the construct in contact with viable bone. Surgical technique was classified as rod alone when no material was placed within the femoral head, as rod + graft when necrotic bone was removed and graft or bone substitute impacted into the resulting cavity, and as rod + biologic when marrow aspirate, cultured cells or a vascularized graft was added.

[a] The lesion was cleared, but no material is described as having been placed. [b] The reamed tract was curetted or scraped, but nothing was placed in the cavity. These three records lie close to the boundary of the classification; the effect of reclassifying them singly and together is reported in Section 3.7 of the main text, and their reclassification removes the statistical significance of the technique contrast.

Lesion size was reported in a recognized classification by two records [31,36] and lesion location by three [18,31,39], in classifications that were not common to them. Both are established prognostic factors that disease stage alone does not capture, and both bear on the mechanism discussed in the main text, since bone ingrowth is expected at the interface with viable bone.
